# H2A.Z Demarcates Intergenic Regions of the *Plasmodium falciparum* Epigenome That Are Dynamically Marked by H3K9ac and H3K4me3

**DOI:** 10.1371/journal.ppat.1001223

**Published:** 2010-12-16

**Authors:** Richárd Bártfai, Wieteke A. M. Hoeijmakers, Adriana M. Salcedo-Amaya, Arne H. Smits, Eva Janssen-Megens, Anita Kaan, Moritz Treeck, Tim-Wolf Gilberger, Kees-Jan Françoijs, Hendrik G. Stunnenberg

**Affiliations:** 1 Department of Molecular Biology, Radboud University, Nijmegen Center for Molecular Life Sciences, Nijmegen, The Netherlands; 2 Bernhard-Nocht-Institute for Tropical Medicine, Hamburg, Germany; 3 M.G. DeGroote Institute for Infectious Disease Research, McMaster University, Hamilton, Ontario, Canada; Albert Einstein College of Medicine, United States of America

## Abstract

Epigenetic regulatory mechanisms and their enzymes are promising targets for malaria therapeutic intervention; however, the epigenetic component of gene expression in *P. falciparum* is poorly understood. Dynamic or stable association of epigenetic marks with genomic features provides important clues about their function and helps to understand how histone variants/modifications are used for indexing the Plasmodium epigenome. We describe a novel, linear amplification method for next-generation sequencing (NGS) that allows unbiased analysis of the extremely AT-rich Plasmodium genome. We used this method for high resolution, genome-wide analysis of a histone H2A variant, H2A.Z and two histone H3 marks throughout parasite intraerythrocytic development. Unlike in other organisms, H2A.Z is a constant, ubiquitous feature of euchromatic intergenic regions throughout the intraerythrocytic cycle. The almost perfect colocalisation of H2A.Z with H3K9ac and H3K4me3 suggests that these marks are preferentially deposited on H2A.Z-containing nucleosomes. By performing RNA-seq on 8 time-points, we show that acetylation of H3K9 at promoter regions correlates very well with the transcriptional status whereas H3K4me3 appears to have stage-specific regulation, being low at early stages, peaking at trophozoite stage, but does not closely follow changes in gene expression. Our improved NGS library preparation procedure provides a foundation to exploit the malaria epigenome in detail. Furthermore, our findings place H2A.Z at the cradle of *P. falciparum* epigenetic regulation by stably defining intergenic regions and providing a platform for dynamic assembly of epigenetic and other transcription related complexes.

## Author Summary

*Plasmodium falciparum* is a unicellular pathogen that is responsible for the most severe form of malaria. Similar to other eukaryotic organisms, its genome is organized into chromosomes by proteins called histones. Modification or replacement of these histones has marked effects on the packaging grade of DNA and instructs the recruitment of protein complexes, thereby regulating essential cellular processes such as gene expression and replication. Here we unveil the genome-wide localization of two histone H3 modifications (K9ac/K4me3) and a histone variant, H2A.Z, during development of the parasite in the human red blood cells. We find that all three epigenetic features are predominantly present in intergenic regions of the *P. falciparum* genome, suggesting an interconnecting role in regulation of gene expression. H2A.Z levels appear to be largely invariable throughout intraerythrocytic development while placement/removal of the histone marks is dynamic with H3K9ac and H3K4me3 being transcription-coupled and stage-specific, respectively. These observations support a model in which H2A.Z-containing nucleosomes serve to demarcate regulatory regions in the parasite's genome and promote transcription initiation by guiding chromatin modifying and transcription initiating complexes. The findings and methodological developments presented in this paper provide a cornerstone for future epigenome research in eukaryotic pathogens and vital information to understand and to interfere with parasite development and survival.

## Introduction

*Plasmodium falciparum*, the deadly protozoan parasite, causes malaria via the invasion of erythrocytes. During its ∼48 h asexual reproductive cycle within human red blood cells (RBC), the parasite exploits and remodels the host cell, multiplies and finally ruptures the RBC to invade fresh erythrocytes. This process demands the timely expression of distinct sets of proteins [1]. Accordingly, analysis of steady-state RNA levels revealed a precise, well-timed program for expression of functionally similar groups of genes [2], [3], [4], while nuclear run-on experiments demonstrated that the bulk of the transcriptional activity occurs at metabolically active and replicative stages of development [5]. Although post-transcriptional mechanisms like mRNA decay [6] and translational repression [7], [8] are involved in the regulation of certain groups of genes, regulatory mechanisms are likely to predominantly act on transcription initiation [9]. Due to the enormous evolutionary diversification of DNA binding proteins, a handful of putative trans-acting factors have only recently been identified [10]. Putative functions of AP2-type DNA binding proteins in a simple cascade of gene activation/repression have been postulated [11] and recently two AP2 proteins were shown to act as essential transcription factors in *P. berghei* ookinete [12] and sporozoite [13] development. However, the hypothesis that a cascade of AP2 factors would regulate the waves of gene expression typical to intraerythrocytic development of *P. falciparum* [11] still needs experimental verification.

In addition to direct regulation of gene expression by DNA binding proteins, posttranslational modification of histones and associated changes in the chromatin structure play a key role in long-term maintenance of the gene expression status. Epigenetic states are established and amended by several distinct mechanisms such as DNA methylation, non-coding RNAs, histone tail modifications, nucleosome remodelling or exchange of histone variants [14]. Intriguingly, the Plasmodium genome is devoid of DNA methylation [15] and lacks the RNA interference machinery [16], although non-coding RNAs [17], [18] and antisense transcripts [19] have been detected. Analysis of Plasmodium histones identified 44 different posttranslationally modified residues and four different histone variants (H2A.Z, H2Bv, H3.3 and CenH3; [20], [21]). Accordingly, histone modifying enzymes and chromatin remodelers appear to be well represented in apicomplexan parasites [22], [23]. With the genome-wide analysis of a few histone modifications/chromatin-associated proteins we have just begun to unveil the unique features of the malaria epigenome [24], [25]. For example, deacetylation and subsequent tri-methylation of lysine 9 on histone H3 as well as recruitment of heterochromatin protein 1 (PfHP1), demarcate heterochromatic islands and are likely mandatory for general silencing of resident antigenic variation genes [24], [26], [27], [28]. About 90% of the *P. falciparum* genes, however, fall outside these H3K9me3/HP1-marked heterochromatic domains. Therefore the malaria epigenome, unlike its human counterpart, is dominated by histone modifications generally associated with transcriptionally active states of genes (i.e. H3K4me3, H3K9ac, H3K14ac and H4ac; [21]). Our exploratory survey revealed a rather unusual pattern of enrichment of two of these marks (H3K4me3 and H3K9ac) in almost all intergenic regions [24] and provided evidence that epigenetic marking is subject to changes during intraerythrocytic development. The exact timing of these changes and the mechanism by which they are targeted to intergenic regions remained enigmatic.

Here we report the genome-wide ChIP-seq profiling of three epigenetic features (H3K4me3, H3K9ac and H2A.Z) as well as RNA-seq analysis of the transcriptome throughout *Plasmodium falciparum* intraerythrocytic development. Importantly, development of a linear amplification method for next generation sequencing was vital for true genome-wide ChIP-seq analysis and enabled, for the first time, highly quantitative analysis of the extremely AT-rich intergenic regions. High-resolution analysis both in space and time was essential to reveal the different dynamics of H3K4me3 and H3K9ac marking. Collectively our findings support the hypothesis that H2A.Z-containing nucleosomes stably demarcate intergenic/regulatory regions of the *P. falciparum* genome and serve as a scaffold for stage-specific as well as transcription-coupled recruitment of histone modifying enzymes that dynamically place, read or erase histone modifications.

## Results

### Novel deep-sequencing approach for analysis of malaria epigenome and transcriptome

**Figure 1:**
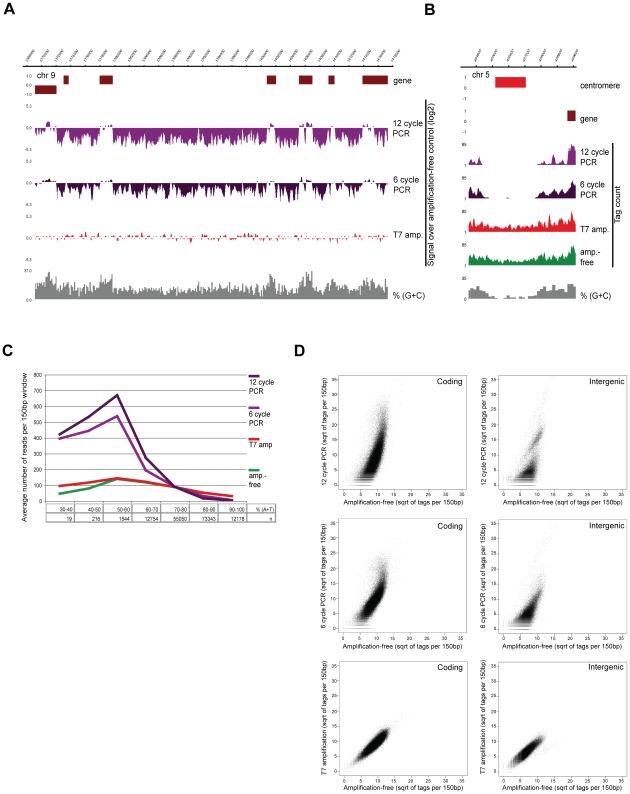
T7 amplification results in perfectly representative libraries for Illumina sequencing. (**A–B**) Screenshots of sequencing data of *P. falciparum* genomic DNA prepared by different amplification methods on (**A**) a part of chr9 (log2-ratio plots of the sequencing data obtained after amplification over amplification-free control) and (**B**) the centromeric regions of chr5 (76 bp sequence reads plotted per 10 bp window) (**C**) distribution of sequence reads in relation to AT-content (“n” refers to the number of 150 bp windows with given AT-content, y-axis displays average number of tags per 150 bp window) (**D**) Scatterplot analysis of amplified sequencing data against amplification-free gold standard in 150 bp windows across GC-richer coding and AT-rich intergenic regions of the *P. falciparum* genome.

In this study we applied state-of-the-art deep-sequencing technology to explore the epigenome (ChIP-seq) and transcriptome (RNA-seq) of *P. falciparum* at multiple stages of intraerythrocytic development (iRBC cycle). Given that analysis of highly AT-rich sequences provided a major challenge and hindered quantitative analysis of the malaria genome and recently published amplification-free method [29] is not applicable for sequencing when small quantities of input material are available, we developed a T7 polymerase-based method for library preparation resulting in unbiased amplification of samples. We tested the new method on sonicated genomic DNA and compared it to the standard PCR-based and amplification-free sample preparation protocol [29]. Visual inspection of the sequencing data revealed marked differences in coverage between the amplification-free and PCR-amplified genomic DNA (Figure 1A). Relative depletion or complete loss at AT-rich intergenic regions was apparent even with just 6 cycles of amplification. On the contrary, mapping of sequences from the T7-amplified library resulted in nearly homogenous coverage indistinguishable from that of the amplification-free control (Figure 1A) even at 100% AT-rich centromeric regions (Figure 1B). To quantify the bias in relation to the DNA sequence composition, we counted the number of sequenced reads mapped to each 150bp window of the Plasmodium genome and plotted these against their AT-content (Figure 1C). As previously reported [29], PCR amplification biases in favour of average GC sequence composition and causes substantial depletion or even complete loss of signal for AT-rich fragments. However, T7-amplified and control libraries display identical sequence coverage. Notably, extremely AT-rich sequences are slightly underrepresented in unamplified as well as in T7-amplified genomic DNA (Figure 1C) probably due to a slight bias in amplification during cluster formation. Biased PCR- and unbiased T7-amplification was confirmed by qPCR (data not shown).

Finally, we determined the correlation of the different amplification methods by plotting the tag counts in 150 bp windows against the unamplified control. As expected, both 12- and 6-cycle PCR-amplified genomic sequences deviate substantially from the amplification-free sequences both in coding and intergenic regions, whereas the correlation between T7 and no amplification is linear (Figure 1D). In conclusion, our method enables preparation of highly representative NGS libraries from few nanograms of starting material, providing an invaluable tool for highly quantitative analysis of extreme genomes/transcriptomes, like that of *P. falciparum.*

Using this optimized protocol in combination with immunoprecipitation of mono-nucleosomal DNA (native ChIP), we generated genome-wide profiles of histone H3 lysine 4 tri-methylation (H3K4me3), histone H3 lysine 9 acetylation (H3K9ac) and histone H2A variant (H2A.Z) at 4 stages (early ring, late ring, trophozoite, schizont) during the iRBC cycle (Figure S1). A minimum of 5.5 M uniquely mapped, 76 bp sequence reads for each sample provides app. 20× overall coverage and over 95% mapability for the *P. falciparum* genome (Figure S2). To correct for nucleosome occupancy, mono-nucleosomal input DNA was also sequenced from the corresponding samples. As an illustration of the effectiveness of the optimized sequencing protocol, highly AT-rich intergenic nucleosomal DNA was readily detected in these input tracks, demonstrating comparable nucleosome occupancy in coding *vs* intergenic regions (Figure S3A) and coverage of nucleosomes over the entire *P. falciparum* genome. This is in contrast to other reports [30], [31], which showed a lack of nucleosomes in intergenic regions (Figure S3B).

Our extensive ChIP-seq dataset provides detailed insight into the dynamics of the epigenome during intraerythrocytic development (discussed below), which confirms and very significantly extends our earlier findings on H3K4me3 and H3K9ac obtained by the use of microarray technology [24]. We verified that both histone H3 modifications are predominantly present at intergenic regions. Furthermore, we detect dynamic marking of intergenic regions during 4 stages of intraerythrocytic development, which significantly expands the analysis performed on the 5′ ends of coding regions from rings and schizonts we reported previously [24]. Additionally, the increased temporal and special resolution unveiled an uncoupling of H3K9ac and H3K4me3 marks in early stages of intraerythrocytic development (see below).

### RNA-seq analysis of the Plasmodium transcriptome during intraerythrocytic development

To correlate epigenetic features with steady state level of mRNAs, RNA-seq was performed at 8 time-points during the iRBC cycle on polyA+ RNA (Figure S1). The obtained sequence data provides deep coverage of the blood-stage transcriptome and evidently contributes to the ongoing refinement of the *P. falciparum* genome annotation. Given the high sensitivity of the method we obtained sequence reads from most coding regions of the Plasmodium genome indicating that over 90% of all genes are expressed to some extent (0.1–50000 tags/1000 bp) during intraerythrocytic development. As expected, the transcription profile of our parasite culture exhibits good general correlation with the recently published *P. falciparum* transcriptome [4] in cycle progression (Figure S4A, R^2^ = 0.73–0.78 for comparable stages). However, correlations between 2 subsequent stages within either of the datasets (representing a 5 or 8 hour stage advance) are higher than those of similar time-points between the datasets. These differences likely result from differences in parasite strain/clone, culturing conditions and synchronicity/staging, stressing that for precise correlation of histone marks to RNA levels it is necessary to generate transcriptome data from the same parasite population used for epigenome analysis.

Normalisation methods presume equal transcriptional activity across stages (i.e. normalise to total signal intensity of arrays or to total RNA-seq tag number). However, transcriptional activity has been shown to significantly vary, being low after invasion of a red blood cell and reaching the highest level at trophozoite stage [5]. We observed this variation in overall transcriptional activity in the RNA yield from these stages (Figure S4B). Therefore, correction of the normalised RNA-seq tag numbers at each stage for the total transcriptional activity per nuclei (see M&M section for details) provides a better approximation of the transcriptional output per gene at each stage. Accordingly we used this correction method when comparing RNA levels with epigenetic features through the intraerythrocytic cycle.

### H2A.Z marks euchromatic intergenic regions

**Figure 2:**
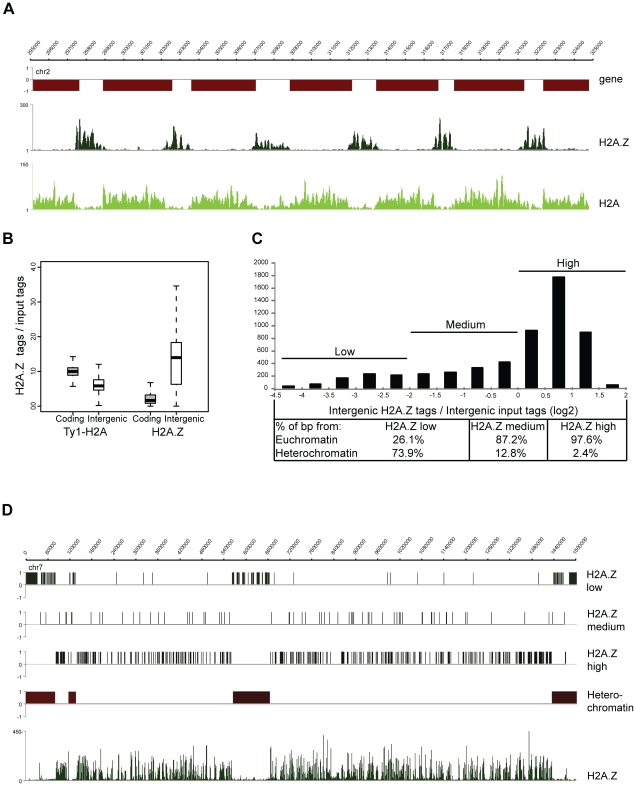
H2A.Z localizes to euchromatic intergenic regions. (**A**) Screenshots of the H2A.Z and H2A-Ty1 ChIP-seq coverage plots obtained from schizont stage parasites (**B**) Box-plot displaying the ratios of ChIP'ed vs mono-nucleosomal input DNA tag counts in every coding and intergenic regions of the *P. falciparum* genome. (**C**) Distribution of H2A.Z over mono-nucleosomal input tag counts in intergenic regions displayed as a histogram and their separation to three categories (low, medium, high). Table lists the percent of basepairs for each category located in heterochromatic vs. euchromatic domains of the *P. falciparum* epigenome. (**D**) Localization of intergenic regions with different H2A.Z occupancy (from C) across entire chromosome 7. H2A.Z coverage plot is included at the bottom.

The *P. falciparum* genome encodes for multiple histone variants (H2A.Z, H2Bv, H3.3, CenH3), but their localization and function has remained elusive. To explore the function of the histone variants we set out to analyze the genomic localization of H2A.Z. Since the N-terminus amino acid sequence of PfH2A.Z is distinct from that of other eukaryotes, an antibody against its N-terminal peptide was raised and proved to recognize both acetylated and non-acetylated forms of the protein (Figure S5). For the analysis of its canonical counterpart, H2A, a parasite line ectopically expressing a Ty1-epitope tagged version of H2A was generated (Figure S6). Using these tools we profiled the genome-wide distribution of H2A.Z- and H2A-containing nucleosomes in schizont stage parasites. This analysis revealed clear enrichment of H2A.Z in intergenic regions of the Plasmodium genome and a largely complementary Ty1-H2A pattern with enrichment in the coding body of genes (Figure 2A). Similar complementary patterns were also apparent when the ChIP-over-input ratio was calculated and plotted for each intergenic and coding region of the genome (Figure 2B). Notably, H2A.Z occupancy differs considerably between intergenic regions (Figure 2B,C). To understand the nature of this variation, we plotted H2A.Z-over-input ratios of intergenic regions in a histogram, divided this into three categories based on their level of H2A.Z occupancy (Figure 2C) and correlated them with different genomic features (Figure 2C,D). Most strikingly, intergenic regions low in H2A.Z were predominantly located at subtelomeric and chromosome internal heterochromatic islands (74%; Figure 2C,D) as defined by the presence of H3K9me3 and HP1 [24], [26]. General depletion of H2A.Z from the heterochromatin domains is also apparent on the chromosome-wide view of its ChIP-seq profile (Figure 2D) and is complemented by an increase of H2A occupancy (data not shown).

In summary, H2A.Z localises preferentially to euchromatic intergenic regions in the *P. falciparum* genome and likely makes these regions more accessible for binding of other proteins. However, contrary to other organisms where H2A.Z occupies a single or a few nucleosomes adjacent to the transcription start site [32], in Plasmodium the average width of the H2A.Z marked regions is 1248 bp (6–8 nucleosomes, data not shown) nearing the average size of the euchromatic intergenic regions (∼1500 bp).

### H3K9ac and H3K4me3 dynamically co-occur with stably H2A.Z marked regions

To compare the localisation of H2A.Z to H3K9ac and H3K4me3 markings we computed the average gene profiles of these features in schizont stage parasite (Figure S7). These profiles showed a very clear colocalisation of the modifications and H2A.Z over the intergenic regions, suggesting that H3K9ac and H3K4me3 are preferentially placed/retained on or next to H2A.Z-containing nucleosomes.

**Figure 3:**
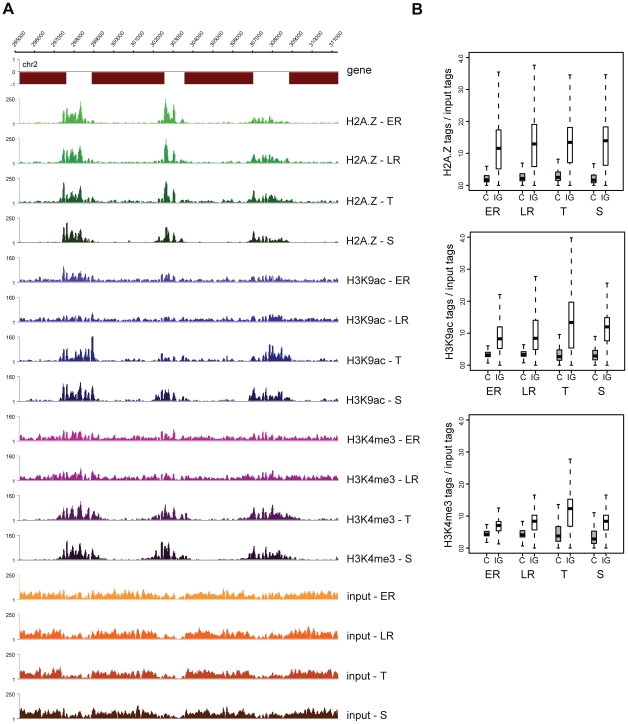
H2A.Z occupancy is invariable, while H3K9ac and H3K4me3 marking is dynamic through the intraerythrocytic cycle. (**A**) Screenshots of the H2A.Z, H3K9ac and H3K4me3 ChIP-seq and mono-nucleosomal input coverage plots at four stages of intraerythrocytic development (ER: early ring; LR: late ring; T: trophozoite; S: schizont) (**B**) Box-plot displaying the ratios of ChIP'ed vs mono-nucleosomal input DNA tag counts in every coding and intergenic region of the *P. falciparum* genome at four stages of intraerythrocytic development.

Performing ChIP-seq analysis on multiple stages throughout the iRBC, we observed that H2A.Z occupancy largely remains constant across stages, while H3K9ac and H3K4me3 levels show clear variation (Figure 3A, S8). This stable H2A.Z level and dynamic H3 marking (peaking at the trophozoite stage) is also apparent when ChIP-over-input ratios are plotted for each intergenic region of the genome (Figure 3B). Together these observations support a hypothesis that in *P. falciparum* H2A.Z demarcates intergenic regions and by itself or in combination with other proteins likely serves as a scaffold for recruitment of histone modifying complexes that acetylate H3K9 and/or trimethylate H3K4.

Visual inspection of the ChIP-seq profiles revealed two exceptions where dynamic H3 marking occurred independent of stable H2A.Z marking. In the coding body of over 100 genes, local increase in H3K9ac was observed specifically at the trophozoite stage (see Figure S9 for a representative example). Markedly, these changes occurred in regions with low H2A.Z levels and were not accompanied by H3K4me3. Given the occurrence of these acetylated islands specifically at the replicative stage and their rather regular spacing (on average 150–200 kb), they might correspond to the origins of replication, which have been shown to be regularly spaced [33] and regulated by H3 and H4 acetylation in other organisms [34], [35], [36], [37] but have not yet been identified in Plasmodium. The mechanism of acetylation of those regions is currently unknown, but a histone acetyl transferase (HAT) has been reported to associate with the origin recognition complex (ORC) in other organisms [38], [39], [40]. Therefore recruitment of acetylases in a replication-coupled manner directly by ORC components might be responsible for the observed acetylation independent of H2A.Z deposition.

Another intriguing example of H2A.Z-independent H3K9ac (and H3K4me3) marking during early stages of the iRBC cycle (Figure S10) can be observed about 2 kb upstream of antigenic variation genes located at the very end of the chromosomes (upsB type *var* genes). *var* genes have been shown to be expressed in a mutually exclusive manner during ring stage [41]. Since multiple *var* genes are active in our parasite population and the ring-stage acetylation is observed at almost all upsB type promoters, it seems that this modification has no direct causal relation with the expression of these genes. It is intriguing however that this marking is specific to 1–2 nucleosomes located directly adjacent to/overlapping with SPE2 repeats (Figure S10), which have been shown to be bound by an AP2-type DNA binding protein, PfSIP2, at least during late stages of development [42]. Therefore, binding of this or other factor(s) associated with the SPE2 motif likely contributes to the dynamic (de)acetylation of these regions.

### H3K9 acetylation follows dynamic changes in transcriptional activity while H3K4 trimethylation is stage-specific

To assess the role of these epigenomic features in relation to gene expression, we first computed the average gene profiles of euchromatic genes with different mRNA levels (low, medium-low, medium-high, high) within each stage and compared them among stages (Figure S11). These profiles indicated general correlation between mRNA expression and H2A.Z deposition as well as H3K9 acetylation at the 5′end and upstream of genes at all stages. Intriguingly, unlike H2A.Z and H3K9ac, H3K4me3 did not exhibit general correlation with transcriptional activity at early stages. Although a higher H3K4me3 marking can be observed on the promoter and 5′ coding sequence of active genes at the latest stages of development (as previously shown by [24]), this increase is largely independent of the level of transcription.

**Figure 4:**
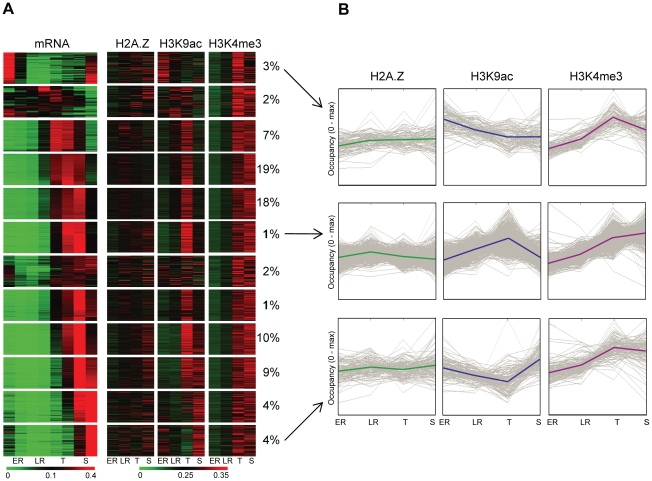
H3K9 acetylation dynamically associates with transcriptional activity during intraerythrocytic development. (**A**) Heatmap representation of the relative transcriptional activity of ∼3800 euchromatic genes and relative H2A.Z occupancy or H3K9ac/H3K4me3 marking upstream of genes throughout intraerythrocytic development (1 equals to the sum of occupancy values at all stages). 12 groups of genes were identified based on their transcriptional profile using k-means clustering. Note that this clustering has been performed after correcting for total transcriptional activity per nucleus (Figure S4). The percent of genes belonging to each cluster are displayed next to the cluster. (**B**) H2A.Z, H3K9ac and H3K4me3 profiles were plotted through development for 3 clusters of genes (from Fig. 4A, indicated by arrows) with maximum transcriptional activity at the beginning (top), in the middle (middle) or at the end (bottom) of the intraerythrocytic cycle. Y-axis was scaled to maximal occupancy. Solid lines represent average H2A.Z, H3K9ac or H3K4me3 profiles for each cluster, grey lines represent individual genes.

Next we investigated whether the increased level of these marks at the promoter of genes follows the changes in gene expression through intraerythrocytic development. We performed k-means clustering of euchromatic genes based on the relative abundance of their transcript across 8 stages (Figure 4). Then, we compared these gene expression profiles with the relative level of H2A.Z, H3K9ac or H3K4me3 occupancy immediately upstream of each gene across 4 stages (Figure 4A). H2A.Z showed relative constant occupancies across these stages, largely independent of changes in gene expression (Figure 4A), supporting our hypothesis that it functions as a ‘stable mark’. On the other hand H3K9ac patterns clearly followed the patterns of expression for most genes (Figure 4A) including genes with highest mRNA level at ring stages. Strikingly, H3K4me3 showed only minor enrichment at early stages but clear marking at later stages at all promoter regions. Dynamic association of H3K9ac with transcriptional activity and invariable increase in H3K4me3 at later stages independent of transcription, is also evident on the average pattern of these features for 3 clusters of early, mid and late expressed genes (Figure 4B).

In summary, our data supports a hypothetical model in which H2A.Z-containing nucleosomes serve as a constant mark of intergenic regions in the *P. falciparum* genome. As such they likely provide a platform for histone modifying enzymes to place and/or remove H3K9ac in a transcription-coupled manner, while H3K4me3 appears to be deposited in a stage-specific manner.

## Discussion

In this study we present detailed genome-wide localization maps and comprehensive analysis of three epigenetic features accompanied by in-depth transcriptome sequencing during the intraerythrocytic development of the protozoan parasite, *Plasmodium falciparum*. Our findings reveal important insights into the dynamic as well as static components of the malaria epigenome and how they contribute to the control of gene expression.

Thus far, genome-wide studies on the Plasmodium epigenome [24], [26], [27], [31], [42], [43] have been performed on microarrays. There are, however, several limitations to this technology; the most serious drawback for its use in malaria epigenomics is the lower and highly variable signal obtained from intergenic probes. To perform highly quantitative epigenome and transcriptome analysis at high resolution, we utilised the technological advances provided by next generation sequencing (NGS) technology. Deep sequencing has many advantages over microarray hybridization [44] but the method is still under intense development and suffers from ‘childhood sicknesses’ [45]. For example PCR amplification, typically used to obtain sufficient amount of DNA results in under-representation or even complete loss of sequences with high AT-content (characteristic of *P. falciparum*), severely hampering the quality and quantitation of the sequence data. Omitting PCR amplification altogether has been reported to circumvent this problem efficiently [29], but it is not feasible for many applications when limited amounts of material is available (ChIP-seq, RNA-seq, GRO-seq, 5C, Ribo-seq, etc). Therefore we developed a linear T7-based method for NGS library amplification that effectively circumvents this bias and yields representative libraries for sequencing. We have unambiguously shown that our method results in unbiased sequencing coverage of the *P. falciparum* genome, and for the first time enables highly quantitative analysis of extremely AT-rich intergenic regions (even up to 100%). Importantly, by using the unbiased protocol to sequence mono-nucleosomal DNA fragments we clearly demonstrate that nucleosome occupancy is comparable between coding and intergenic regions of the *P. falciparum* genome (Figure S3A). Therefore we assume that the recently reported low intergenic signal in histone H4 ChIP-on-chip [31] or MAINE-seq [30] experiments is likely due to a general loss of AT-rich sequences during sample preparation, rather than to lower nucleosome occupancy, because their “nucleosomal occupancy” closely follows the genomic GC-content (Figure S3B). Furthermore, we detect nucleosomes throughout the entire *P. falciparum* genome, but we cannot exclude the existence of local and temporal nucleosome free regions.

Our ChIP-seq analysis shows that euchromatic intergenic regions of the *P. falciparum* genome are occupied by a specific nucleosome subtype that contains the histone variant H2A.Z. This variant in other organisms has been reported to have diverse functions, including heterochromatin formation and boundary maintenance, but is most commonly associated with the transcription start site of a subset of genes and involved in transcription initiation [46]. As H2A.Z has been found at the 5′ end of both active and inactive genes in yeast [47] it is argued that it provides an open or poised chromatin environment that is amendable both for transcriptional activation or repression. In *P. falciparum* we find that H2A.Z is stably present at both 5′ and 3′ ends of euchromatic genes, thus it serves as a constant feature of intergenic regions. Although H2A.Z occupancy appears to be invariable across the four developmental stages its level is highest in the promoter region of genes with the highest mRNA level (Figure S11). Therefore it might define the general strength of promoters but not their temporal activity. Such transcription promoting/permissive function may be exerted by the physicochemical properties of this nucleosome subtype. H2A.Z, in combination with other variants (H3.3 [48] or H2Bv [49], [50]) has been reported to form less stable nucleosomes and likely facilitates the access of transcription factors to the underlying DNA sequence thereby promoting transcription. Notably, acetylation of H2A.Z specifically occurs at promoters of active genes in yeast [51] and probably further facilitates an “open” chromatin state. Since our antibody recognizes both acetylated and non-acetylated H2A.Z with similar affinity, further experiments with acetylation specific antibody will be required to characterise the distribution of acetylated H2A.Z in the malaria epigenome. Previous mass-spectrometric analyses however provided a strong indication that unlike its metazoan counterpart [52], [53] the chromatin-associated H2A.Z pool in *P. falciparum* [21] is dominated by the acetylated form, suggesting a general transcriptionally permissive function. Orthologs of the histone acetyl transferases (Gcn5 and Esa1) that acetylate the N-terminus of H2A.Z in higher eukaryotes [51] have been found in Plasmodium [10]. Intriguingly, the amino acid sequence of this histone tail differs substantially between the parasite and its human host (Figure S5). Therefore it is tempting to speculate that besides or instead of Gcn5 and Esa1 orthologs, any of the seven less conserved HATs encoded in Plasmodium genome might be involved in acetylation of the unique N terminal extension of PfH2A.Z.

In yeast, deletion of H2A.Z leads to decreased expression of genes close to the subtelomeric heterochromatic domain suggesting a function of H2A.Z in boundary maintenance [54]. This could also be the case in Plasmodium. Selective deposition of H2A.Z to euchromatin, or its eviction from heterochromatin could be guided by marks of other core histones characteristic to these regions (reviewed in [55], [56]). For example in yeast, several acetylation marks (e.g. H3K14ac [57], [58]; or H4K16ac [59]) and a bromodomain containing component of the SWR1 complex have been implicated in targeted H2A.Z deposition [57], [58]. Such mechanism could also be responsible for the higher H2A.Z occupancy of active genes in Plasmodium but an additional sequence based mechanism might exist to fully confine H2A.Z to intergenic regions of the malaria genome. One such mechanism has been described in yeast, where a 22bp long sequence motif commonly found in promoters is necessary and sufficient for H2A.Z deposition and formation of adjacent nucleosome-free region [47].

Finally, H2A.Z could promote transcription by recruitment of histone modifying/remodelling complexes. The almost perfect colocalisation of H2A.Z with H3K4me3 and H3K9ac in intergenic regions of the Plasmodium genome (Figure S7) raises the intriguing hypothesis that these nucleosomes indeed serve as a preferred substrate for H3 modifying enzymes and that the H2A.Z-marked intergenic regions provide a scaffold for the dynamic placement/removal of specific histone marks. The exact composition of this H2A.Z-containing nucleosome subtype is at the moment unknown and any other histone (variant) or histone modification contained within this nucleosome subtype might be responsible (alone or in combination) for the recruitment of histone modifiers/chromatin remodelers. However, similar enrichment of H3K4me2–3 and H3K9ac on H2A.Z-containing nucleosomes was observed in other eukaryotes [60], [61] suggesting that the underlying mechanism is evolutionary conserved. Notwithstanding these observations and the fact that similar crosstalk between other core histones has been described (e.g. H2A and H4 [62] or H2B and H3 [63]), the proteins involved in H2A.Z-containing nucleosome dependent recruitment of histone methyltransferases or acetylases remain to be identified.

Similar to H2A.Z, H3K9ac is also a common feature of euchromatic intergenic regions in the Plasmodium genome as most promoters and even the 3′ end of genes are acetylated to some extent as compared to coding bodies of genes (as reported previously by us [24] and others [31]). However, in contrast to the stable H2A.Z occupancy throughout the developmental stages, the level of H3K9ac at promoter regions dynamically follows the pattern (Figure 4) and level (Figure S11) of transcription throughout intraerythrocytic development. Similar association of this mark with transcriptional activity has been observed in yeast and higher eukaryotes [64], [65]. The temporal resolution of our dataset does not allow firm conclusions whether H3K9 acetylation occurs before or after transcriptional activation and thus is required for transcription or simply is a consequence of it. Notably, the changes in H3K9ac levels between different stages are moderate (2.1 fold on average) and may not solely instruct stage-specific transcription. Interestingly, Chaal and colleagues (2009 [43]) observed dramatic changes in gene expression upon treating malaria parasites with an HDAC inhibitor (apicidin), but found no direct link between changes in acetylation levels and transcription. Similarly, we find that treatment of parasites with another HDAC inhibitor (SAHA) affects the acetylation level of a large number of genes in the parasite's genome, but leads to the activation of only a small group of genes (Salcedo-Amaya and Stunnenberg, unpublished observations). Collectively these observations suggest that although H3K9 acetylation is intimately coupled to transcription in *P. falciparum* it is not or not the only instructive mark. Further studies of the enzymes (complexes) responsible for placement/removal of these marks and their mechanisms of recruitment are indispensable to elucidate the order of events and the underlying mechanism.

Unlike H3K9ac, the dynamic H3K4me3 marking does not follow the temporal pattern of gene expression. Instead H3K4 methylation at nearly all intergenic regions occurs in a stage-specific manner, being low at early stages and increasing at later stages of development (Figure 4). Since we readily detected H3K4 trimethylation over the SPE2 sites (Figure S10) at early stages, we are confident that the lower level of H3K4me3 at ring stages is not due to the lower efficiency of the ChIP. We can, however, not formally exclude the possibility that other modifications on the H3 histone tail (e.g. phosphorylation of T3) interfere with H3K4me3 antibody binding at early stages. Nevertheless, reduced global level of H3K4me3 at early stages has also been observed on Western-blot [66]. The observed stage-specific methylation patterns can be the result of placement of the mark at the trophozoite stage and/or by its removal at the end of the intraerythrocytic development. Histone methylation marks can be placed by SET domain containing enzymes [67]. Of the 9 SET-domain proteins encoded in the Plasmodium genome, PfSET1 (PFF1440w) and PfSET4 (PFI0485c) were predicted to have H3K4 methylation activity based on their homology to yeast and human enzymes [66]. The most likely candidate to remove methylation from H3K4 is the homologue (PFL0575w) of the human demethylases LSD1. Because at the mRNA level none of these enzymes exert distinctive stage specificity ([66], our RNA-seq data), it seems likely that their potentially stage-specific expression/activity is regulated at the translational or posttranslational levels.

Intriguingly, we find H3K4me3 to be enriched at the promoter of active genes at later stages of development independent of their expression levels (Figure S11). As the average gene profile of H2A.Z in schizont stage is identical to that of H3K4me3 (Figure S11), and H3K4me3 marks are found preferentially on regions occupied by H2A.Z (Figure S7), this binary H3K4me3 pattern could simply be the consequence of H2A.Z occupancy.

Although the exact function of H3K4me3 is poorly characterized, this mark has been linked to transcription initiation, elongation and RNA processing in other eukaryotes [68] and was found to associate with several active transcription-related factors including TFIID [69]. Accordingly, H3K4me3 not only colocalizes with H3K9ac but also exhibits a similar function. Since in *P. falciparum* H3K4me3 is present at the intergenic regions at the transcriptional most active trophozoite and schizont stages, it is not unthinkable that it functions in combination with H3K9ac to enhance the expression of those genes that are activated by transcription factors. Indeed, the presence of both H3K4me3 and H3K9ac marks strongly enhanced TFIID binding to histone H3 in mammals [69], indicating that the presence of both marks has a cumulative effect on the recruitment of the transcription machinery. Nonetheless, the disconnect between H3K4me3 and H3K9ac observed in Plasmodium, opens up the appealing possibility that an evolutionary diversification of their functions has occurred in the Plasmodium lineage providing a potential weak spot to specifically target malaria parasites.

In summary our data identifies H2A.Z as a rather static component of the *P. falciparum* epigenome, which demarcates intergenic regions and is part of a scaffold of H2A.Z-containing nucleosomes for subsequent dynamic modification of H3K4 and H3K9 residues in either a stage-specific or transcription-coupled manner. These results set the gateway towards uncovering the role of histone code in indexing the malaria epigenome, in transcriptional regulation and other essential processes in *Plasmodium falciparum,* which might disclose a whole new repertoire of targets for therapeutic intervention.

## Materials and Methods

### Parasite culture

Parasites were cultured in standard RPMI medium supplemented with 10% human serum and 0.2% NaHCO3 in 250 ml tissue culture flasks and candle jars. The culture was synchronised with multiple rounds of sorbitol treatments and Percoll gradient centrifugation. After the last Percoll gradient centrifugation, 5% red blood cells were added with a couple of hours delay ensuring better synchronicity and lack of invasion of fresh RBC before time-point zero (0 hpi). Medium was changed at every 10 hours, but not less than 10 h before collection. After 20 h post-invasion (hpi), double volume of medium was added to ensure optimal development of the parasites. The culture was divided to separate culture flasks (20 ml each) but these were mixed with every change of medium. Samples for RNA and chromatin isolation were collected at every 5 or 10 hpi, respectively (Figure S1). Parasites were immediately placed and processed on ice. At each time point parasitemia, stages and average number of nuclei were defined by visual inspection of Giemsa stained blood smears (Figure S1).

All experiments were carried out on 3D7 *P. falciparum* parasites. For profiling of canonical H2A, parasites were transfected with a pARL-1a- plasmid [70] encoding Ty1-tagged version of PfH2A (PFF0860c) under the control of the chloroquine resistance transporter promoter (Figure S6A). Expression of the transgene in the presence of 40 nM WR99210 was confirmed using the monoclonal anti-TY1 antibody (BB2) that exclusively recognises the tagged H2A protein on Western blot and immune fluorescence microscopy (Figure S6B,C; IFA on methanol fixed parasites were performed according to [71]).

### Generation of polyclonal H2A.Z antibody

The H2A.Z antibody (PFC0920w) was created by immunizing rabbits with the peptide ATA-KVIGGKVGGKVGG conjugated to keyhole limpet cyanin (KLH). Antibody was purified by affinity chromatography using the same peptide. The specificity of the antibody was tested in dotblot with the same as well as an acetylated version (K6 and K10) of this peptide and in Western blot against total nuclear extract from *P. falciparum* mixed stage parasites (Figure S5).

### Ethical statement

The immunization of the rabbit for H2A.Z antibody production used in this study was carried out in strict accordance with the regulations and recommendations of the Dutch Law for Animal Experimentation (WOD). The immunization experiment was carried out at the Central Animal Laboratory of the Radboud University Medical Center and was approved by the Committee for Animal Experiments of the Radboud University (Permit Number: KUNDEC2001-83). All efforts were made to minimize suffering of the rabbit during immunization and the final bleeding was carried out under hypnorm anesthesia.

### Genomic DNA extraction and fragmentation

Genomic DNA was extracted from *in vitro* cultured ring stage malaria parasites (*P. falciparum*, 3D7 strain). Nuclei were isolated after saponin lysis of the red blood cells (0.05% saponin in PBS) and lysis of the parasites (10 mM Tris pH 8.0, 3 mM MgCl2, 0.2% NP40) on a 0.25 M sucrose cushion. Following Proteinase K digestions at 37°C for 4 h (50 ug/ml PK in 10 mM Tris pH 8.0, 20 mM EDTA, 0.5% SDS) remaining proteins were removed by phenol-chlorophorm extraction and genomic DNA was precipitated in the presence of EtOH (2.5 volume) and sodium-acetate (0.1 volume of 3 M). DNA pellets were dissolved in TE (50 ng/ul) and sonicated to fragments of 100–700 bp in 100 ul aliquots using the Bioruptor UCD200 (Diagenode).

### Chromatin immunoprecipitation

Native ChIP was carried out as described earlier [24], [26]. In short: After saponin lysis of the red blood cells and lysis of the parasites, nuclei were separated using a 0.25 M sucrose buffer cushion. Native chromatin was prepared by MNase digestion and subsequent extraction with salt-free buffers (10 mM Tris pH 7.4, 1 mM EDTA; 1 mM Tris pH 7.4, 0.2 mM EDTA). Chromatin was diluted in 2×ChIP incubation buffer (100 mM NaCl, 20 mM Tris pH 7.4, 6 mM EDTA, 1% Triton X-100, 0.1% SDS). ∼400 ng DNA-containing chromatin was incubated with 1 µg antibody (H3K9ac (Diagenode pAb-004-050, lot#DA-0010); H3K4me3 (AbCam ab8580, lot# 617802)) overnight at 4°C followed by the addition of 10 µl A/G beads and further incubation for 2 h. After washing with buffers containing 100, 150 and 250 mM NaCl, immuno-precipitated DNA was eluted and purified using PCR purification columns (Qiagen). For each antibody 3 ChIP reactions were performed in parallel to obtain sufficient amount of DNA for ChIP-seq.

### RNA isolation and cDNA synthesis

RNA was isolated from 20 ml synchronous culture (see *Parasite Culture*) at 8 time-points covering all stages of the intraerythrocytic development. RNeasy Mini Kit (Qiagen) was used according to manufacturer's instructions (12 columns were used for RNA isolation of each sample and the resulting RNA was further cleaned on two columns). DNase treatment was performed on the columns (Qiagen, RNase-Free DNase set) at both rounds of purification. Total RNA was directly subjected to a single round of polyA-selection (Qiagen, Oligotex mRNA Mini Kit) to enrich for mRNAs. Subsequently, 3–5 µg total RNA-equivalent polyA+ mRNA was fragmented by hydrolysis in 40 mM Tris-Acetate pH 8.2, 100 mM potassium acetate, 30 mM magnesium acetate for 1 min 45 sec at 85°C followed by purification via precipitation.

cDNA was synthesized from 3–5 µg total RNA-equivalent fragmented polyA+ mRNA. First strand synthesis was performed by Superscript III Reverse Transcriptase (400 units, Invitrogen) in the presence of 1× first strand buffer (Invitrogen), 11 µg AT-corrected nonamer primers (76%AT), 10 mM DTT, 1 mM each of dNTPs and 40 units RNasin Plus RNase inhibitor (Promega) in 20 µl total volume. The reaction was incubated at 37°C for 10 minutes and subsequently at 42°C for 1 hour. A similar reaction lacking reverse transcriptase (RT- control) was assembled to check for any genomic DNA contamination by qPCR and was negative for all samples.

Single-stranded cDNA was converted to double-stranded cDNA by *E. coli* DNA Polymerase I (40 units, Invitrogen) and *E. coli* DNA ligase (10 units, New England Biolabs) in the presence of RNase H (2 units, Ambion). The 150 µl reaction mixture in 1× second strand buffer (Invitrogen) supplemented with 0.2 mM each of dNTPs was incubated at 16°C for 2 hours. Afterwards, ds cDNA was purified using MinElute Reaction Clean-up Kit (Qiagen). ∼40 ng of each sample ranging in size from 100 bp–1 kb was used for sequencing sample preparation as described below.

### High throughput sequencing

To avoid any changes in the composition of the samples due to PCR amplification we developed an unbiased linear T7 amplification method that produces highly representative sequencing libraries (detailed protocol has been submitted to Nature Protocols and available upon request). In short: End repaired and A-tailed DNA fragments are ligated with custom adapters (A and B) corresponding to the two strand of the Illumina adapters, one of which (B) is extended by the T7 promoter sequence. Ligated and size selected DNA fragments are then *in vitro* transcribed using T7 polymerase. cDNA synthesis from the resulted RNAs is initiated from a primer complementary to the adapter A ensuring that the library will only contain full-length fragments with two distinct adapters. After removal of excess primers via gel extraction, DNA fragments are directly used for cluster formation and sequencing.

To test the newly developed linear amplification protocol, 40 ng of the sonicated genomic DNA was used for library preparation by standard PCR-based (Illumina) or our T7 amplification protocols, and 400 ng DNA was processed by the amplification-free method [29]. Amplification-free samples were processed as 10 reactions of 40 ng.

For ChIP-seq or RNA-seq analysis 6–40 ng immunoprecipitated and mono-nucleosomal input DNA or ds cDNA was used for preparation of sequencing libraries by the T7 amplification method.

Sequencing libraries (32.5 pmol each) were loaded on the Illumina Genome Analyzer IIx and sequenced for 76 cycles from one side of the fragments (Standard Cluster Generation Kit v2 and 2×36-cycle sequencing kit v3). Quality filtered 76 bp sequence reads were mapped against the *Plasmodium falciparum* genome assembly (PlasmoDB v6.1) for ChIP and input samples or against the *Plasmodium falciparum* annotated transcripts (PlasmoDB v6.1) for mRNA samples using BWA [72].

### Data analysis

For comparative analysis a common number of uniquely mapped reads were picked for each dataset, respectively: 10 M for genomic DNA; 5.5 M for ChIPed; 11 M for input, 8.2 M for mRNA samples. (Since for the later ring H3K4me3 ChIP only 3.9 M tags were obtained, for this particular dataset the number of tags was adjusted to 5.5 M by 1.6 M tags randomly selected from the same dataset.).

Coverage plots were generated by counting the number of overlapping, uniquely mapped 76 bp tags in 10 bp windows and visualized in SignalMap (NimbleGen). Ratio tracks were obtained by dividing the tag counts with the corresponding tag counts of the amplification-free method, smoothened by moving average method (in a 150 bp window) and displayed on a log2 scale. GC-track was generated by plotting the GC-content (%) per 150 bp window of the *Plasmodium falciparum* genome (PlasmoDB v6.1).

For scatter plots the number of uniquely mapped tags were counted in 150 bp windows (based on the location of the first base) and plotted against the corresponding value of the amplification-free sample. These 150 bp windows were assigned to coding and intergenic regions (genome annotation PlasmoDB v6.1) if more than half of their sequence falls into either of these categories. To correlate the coverage with AT-content, these 150 bp windows have been grouped on the bases of their AT-content and the average number of unique and non-unique randomly placed tags mapping to these groups was displayed.

To calculate theoretical mapability, all possible sequence tags of a given length that can be obtained from the *P. falciparum* genome (PlasmoDB v6.1) were determined and mapped against this reference genome. Sequence tags at the size of 10–40, 42, 44, 46, 48, 50, 52, 60, 70, 76, 80, 90, 100, 125 and 150 bases were generated using a python script that uses all positions in the genome sequences as a start and takes the given number of bases (length) including and upstream of the starting base. Mapability at each position of the genome was calculated by counting the number of overlapping, uniquely mapped tags in a 10 bp window and plotted as a percent of maximum. For general mapability the percentage of uniquely mapable tags in the whole genome were defined and plotted according to their read length. To determine mapability in subsections of the genome (heterochromatin, euchromatic, euchromatic coding, euchromatic intergenic) tags were assigned to the specified regions if at least 50% of the tag falls within these regions (intersections were done using a Perl script).

Boxplots were generated in R from the ratios of the tag counts in the ChIPed vs input or input vs genomic DNA datasets using standard settings. Tags were assigned to the coding or intergenic regions (genome annotation PlasmoDB v6.1) if more than 50% of the tag falls within these regions. Heterochromatic regions were defined based on genome-wide localization of *Plasmodium falciparum* heterochromatin protein 1 (PfHP1) published by Flueck *et al.* [26]. Intergenic regions were defined as the non-coding sequences between two adjacent genes.

For comparison of different datasets on nucleosome occupancy raw MAINE-seq data for three stages [30] was downloaded from the GEO database and analysed as described above, while processed datafiles for H4 ChIP-on-chip experiments [31] were obtained from PlasmoDB.

To determine correlations between RNA-seq data from Otto and colleagues [4] and our dataset, 36 bp single-end reads (resulting from truncating longer reads to the first 36 bp) were mapped against the *P. falciparum* transcriptome (annotated transcripts PlasmoDB v6.1) and 1.9 M uniquely mapped tags were randomly selected from each. The sum of all tags mapping to one gene was determined for each dataset and correlations were obtained in R on log-transformed values using default settings.

Average gene profiles were computed for ∼2600 genes within the euchromatic domain (given the general lack of signal in the heterochromatic domain) with coding body of 1–10 kb (excluding extremely small or large genes) and a minimum of 800 bp intergenic regions on both sides (to minimize overlap with flanking region of nearby genes). The coding body of these genes was divided into twenty equal size windows and four 150 bp windows immediately up- or downstream represented flanking sequences. The ratio of the tag counts in the ChIPed *vs* input datasets has been computed in each individual windows and averaged in the corresponding window of all genes with similar expression levels (genes has been divided to four equal sized group with different expression levels based on the number of RNA-seq tags mapping to every 1000 bp of their transcript in a given stage).

For expression profiling of genes, RNA-seq sequence reads from 8 stages of the RBC cycle were mapped against the transcriptome (annotated transcripts; PlasmoDB v6.1) and 8.2 M uniquely mapped tags were randomly selected from each. To provide better approximation of the true transcriptional activity, tag density (per 1000 bp) values at each stage were adjusted by a scaling factor derived from total RNA yield and the average number of nuclei, respectively (see Figure S4, scaling factor calculated by dividing total RNA yield by the average number of nuclei per parasite). Relative transcriptional activity of each euchromatic gene (with min 100 tags/1000 bp at least in one of the stages on non-scaled tag counts, including ∼3800 genes in total) was calculated by dividing the scaled tag density at the particular stage by the sum of the scaled tag densities across all 8 stages. Twelve groups of genes with similar expression pattern through the intraerythrocytic cycle were identified by k-means clustering of genes and visualised on a heatmap (MultiExperimentViewer). The expression profile of each gene was then matched with the profile of H2A.Z occupancy/H3K9ac or H3K4me3 marking in the H2A.Z occupied region (peak) immediately upstream of the gene. (H2A.Z peaks were called on the combined H2A.Z ChIP-seq dataset if the ratio over input was higher than 1 on a minimum length of 150 bp). Relative marking/occupancy through the intraerythrocytic cycle was calculated similarly to that of the relative transcriptional activity (scaled to the sum of ChIP-over-input ratios across stages).

### Data deposition

Raw sequencing data and processed data files described in this publication are deposited in NCBI's Gene Expression Omnibus and are accessible through GEO Series accession number GSE23787 for mono-nucleosomal inputs, H2A, H2A.Z, H3K9ac and H3K4me3 ChIP-seq and RNA-seq expression data (http://www.ncbi.nlm.nih.gov/geo/query/acc.cgi?acc=GSE23787). All data is also available at PlasmoDB (www.plasmodb.org).

## Supporting Information

Figure S1 Sample collection scheme. (A) Collection scheme for total RNA (every 5h) and nuclei (every 10h) from highly synchronous *P. falciparum* culture (3D7 strain). hpi: hours post-invasion (B) Representative image of Giemsa stained blood smear at each time point. Percentage of ring (R), trophozoite (T) or schizont (S) stage parasites and the number of nuclei per parasite have been defined by visual inspection of 1000 infected red blood cells.
(5.54 MB PDF)

Figure S2 Mapability of sequences reads with different length on the highly AT-rich *Plasmodium falciparum* genome. (A-C) *Plasmodium falciparum* genomic sequence (PlasmoDB v6.1) was used to generate all possible reads of fixed length. Reads were mapped back onto the genome and the percentage of the uniquely mapable fragments (mapping to a unique position in the genome) were plotted according to the length of the sequence tags in the (A) whole genome (B) euchromatic or repeat-rich and multi gene family containing heterochromatic domains (C) euchromatic coding and AT-rich intergenic regions, respectively. (D-E) Representative screenshot displaying the percentage of uniquely mapable fragments of 36bp, 52bp, 76bp and 100bp in the heterochromatic (D) and euchromatic (E) domains.
(1.40 MB PDF)

Figure S3 Nucleosome occupancy is similar between coding and intergenic regions. (A) Box-plot displaying the ratios of mono-nucleosomal vs genomic DNA tag counts in every coding (C) and intergenic (IG) region of the *P. falciparum* genome at four different stages of intra-erythrocytic development (ER: early ring; LR: late ring; T: trophozoite; S: schizont). (B) Representative screenshot showing direct comparison of H4_ChIP-on-chip (log2-ratio ChIP-over-genomic DNA) [31], MAINE-seq [30] and our mononucleosomal Illumina sequencing coverage plots at three stages of intra-erythrocytic development (ER/R: early ring/ring; T: trophozoite; S: schizont). Bottom track displays GC-content (%) per 150bp window.
(0.72 MB PDF)

Figure S4 RNA-seq interdataset comparison and scaling method. (A) R2-correlation values from pair-wise comparison of our 8 stages RNA-seq and 7 stages of published RNA-seq data [4] of 3D7 *Plasmodium falciparum* parasites. Colour code indicates the level of correlation (black  =  highest, white  =  lowest). (B) Graph showing the scaling factor used for scaling of RNA-seq data to better approximate transcriptional activity per nucleus. Scaling factor is calculated based on the amount of total RNA collected from each stage, divided by the average number of nuclei per parasite in that stage.
(0.23 MB PDF)

Figure S5 Generation and characterisation of polyclonal H2A.Z antibody. (A) Alignment of the protein sequence of histone H2A and H2A.Z proteins from *H. sapiens, P. falciparum, T. brucei, T. gondii*. The highly conserved core domain is surrounded by a dashed line. Acetylated lysines at the N-terminus of H2A.Z are bolded and underlined. The peptide used for immunisation is highlighted in yellow. (B) Dot blot experiment demonstrating similar affinity of the H2A.Z antibody to the acetylated and non-acetylated peptide. (C) The H2A.Z antibody specifically recognises its target protein in total nuclear isolate from asynchronous parasite culture.
(0.91 MB PDF)

Figure S6 Generation and characterisation of H2A-Ty1 strain. (A) Map of the pARL-1a-Ty1-H2A plasmid transfected into 3D7 parasites. (B) Western blot demonstrating specific recognition of the Ty1-tagged H2A protein by the BB2 antibody in the transgenic parasite line. (C) Immunofluorescent assay showing nuclear localisation of the ectopically expressed Ty1-H2A protein at all stages of the intra-erythrocytic cycle.
(0.95 MB PDF)

Figure S7 H3K9ac and H3K4me3 shows very similar localisation to H2A.Z. Average profile of H2A.Z occupancy, H3K9ac and H3K4me3 marking over euchromatic genes in schizont stage parasites. The coding region is indicated by the arrow (divided into 20 bins), 5′ and 3′ intergenic regions are displayed as 4 blocks of 150bp.
(0.17 MB PDF)

Figure S8 H2A.Z occupancy is invariable, while H3K9ac and H3K4me3 markings are dynamic through the intra-erythrocytic cycle. Screenshot of same chr2 region as shown in Figure 3. Instead of coverage plots, H2A.Z-, H3K9ac- and H3K4me3 over mono-nucleosomal input log2-ratios are displayed at four stages of intra-erythrocytic development (ER: early ring; LR: late ring; T: trophozoite; S: schizont).
(0.49 MB PDF)

Figure S9 Regions specifically acetylated on H3K9 independent of H2A.Z during trophozoite stage, might corresponds to origins of replication. Screenshot of the H2A.Z, H3K9ac and H3K4me3 ChIP-seq coverage plots at four stages of intra-erythrocytic development (ER: early ring; LR: late ring; T: trophozoite; S: schizont) from a subsection of chromosome 10. The region with increased H3K9ac only in trophozoite stage is surrounded by a dashed line.
(1.53 MB PDF)

Figure S10 SPE2 repeats upstream of upsB type var genes carry H3K9ac and H3K4me3 independent of H2A.Z. Screenshot of the H2A.Z, H3K9ac and H3K4me3 ChIP-seq coverage plots at four stages of intra-erythrocytic development (ER: early ring; LR: late ring; T: trophozoite; S: schizont) from the distal end of chromosome 2. SPE2 repeat containing region carrying H3K9ac and H3K4me3 during ring stage is surrounded by a dashed line.
(1.13 MB PDF)

Figure S11 H2A.Z occupancy and H3K9ac marking upstream of genes correlates with steady state mRNA levels. Average profile of H2A.Z occupancy, H3K9ac and H3K4me3 marking over groups of genes with different mRNA levels at four stages of intra-erythrocytic development. To avoid signal coming from the neighbouring genes, only euchromatic genes (∼2600) with a minimum of 800bp intergenic region on both sides were included in this analysis. Coding body is indicated by the arrow (divided into 20 bins), 5′ and 3′ intergenic regions are displayed as 4 blocks of 150bp.
(0.72 MB PDF)
