# Supplementary material for: H2A.Z Demarcates Intergenic Regions of the Plasmodium falciparum Epigenome That Are Dynamically Marked by H3K9ac and H3K4me3
Source: PLoS Pathog. 2010 Dec 16;6(12):e1001223. doi: 10.1371/journal.ppat.1001223 (PMC3002978; doi:10.1371/journal.ppat.1001223)
Supplement: Figure S1 — Sample collection scheme. (A) Collection scheme for total RNA (every 5h) and nuclei (every 10h) from highly synchronous P. falciparum culture (3D7 strain). hpi: hours post-invasion (B) Representative image of Giemsa stained blood smear at each time point. Percentage of ring (R), trophozoite (T) or schizont (S) stage parasites and the number of nuclei per parasite have been defined by visual inspection of 1000 infected red blood cells. (5.54 MB PDF) [file ppat.1001223.s001.pdf]

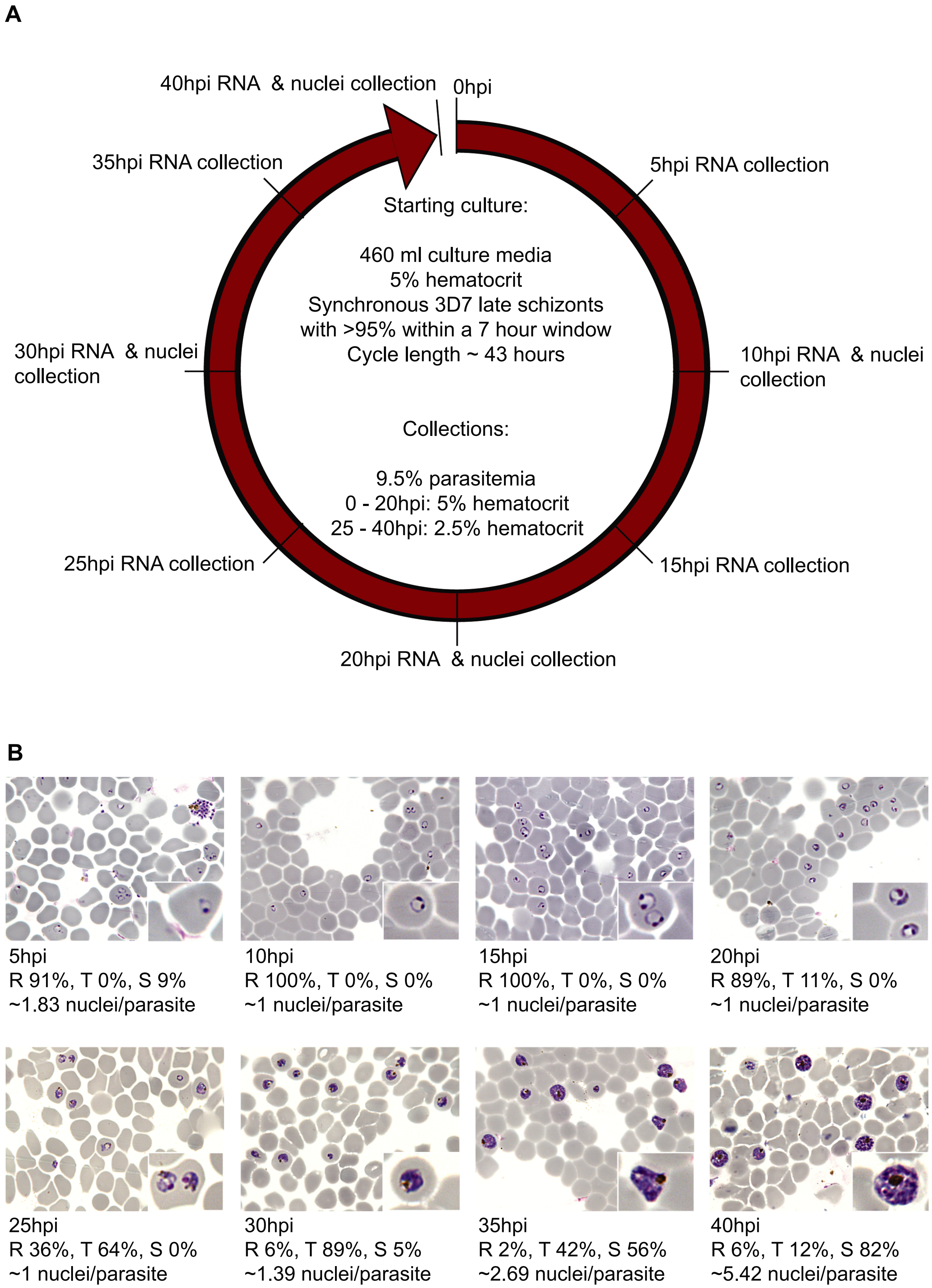
A
40hpi RNA & nuclei collection
0hpi
35hpi RNA collection
5hpi RNA collection
Starting culture:
460 ml culture media
5% hematocrit
Synchronous 3D7 late schizonts
with >95% within a 7 hour window
Cycle length ~ 43 hours
30hpi RNA & nuclei collection
10hpi RNA & nuclei collection
Collections:
9.5% parasitemia
0 - 20hpi: 5% hematocrit
25 - 40hpi: 2.5% hematocrit
25hpi RNA collection
15hpi RNA collection
20hpi RNA & nuclei collection
B
5hpi
R 91%, T 0%, S 9%
~1.83 nuclei/parasite
10hpi
R 100%, T 0%, S 0%
~1 nuclei/parasite
15hpi
R 100%, T 0%, S 0%
~1 nuclei/parasite
20hpi
R 89%, T 11%, S 0%
~1 nuclei/parasite
25hpi
R 36%, T 64%, S 0%
~1 nuclei/parasite
30hpi
R 6%, T 89%, S 5%
~1.39 nuclei/parasite
35hpi
R 2%, T 42%, S 56%
~2.69 nuclei/parasite
40hpi
R 6%, T 12%, S 82%
~5.42 nuclei/parasite
