# Supplementary material for: H2A.Z Demarcates Intergenic Regions of the Plasmodium falciparum Epigenome That Are Dynamically Marked by H3K9ac and H3K4me3
Source: PLoS Pathog. 2010 Dec 16;6(12):e1001223. doi: 10.1371/journal.ppat.1001223 (PMC3002978; doi:10.1371/journal.ppat.1001223)
Supplement: Figure S2 — Mapability of sequences reads with different length on the highly AT-rich Plasmodium falciparum genome. (A-C) Plasmodium falciparum genomic sequence (PlasmoDB v6.1) was used to generate all possible reads of fixed length. Reads were mapped back onto the genome and the percentage of the uniquely mapable fragments (mapping to a unique position in the genome) were plotted according to the length of the sequence tags in the (A) whole genome (B) euchromatic or repeat-rich and multi gene family containing heterochromatic domains (C) euchromatic coding and AT-rich intergenic regions, respectively. (D-E) Representative screenshot displaying the percentage of uniquely mapable fragments of 36bp, 52bp, 76bp and 100bp in the heterochromatic (D) and euchromatic (E) domains. (1.40 MB PDF) [file ppat.1001223.s002.pdf]

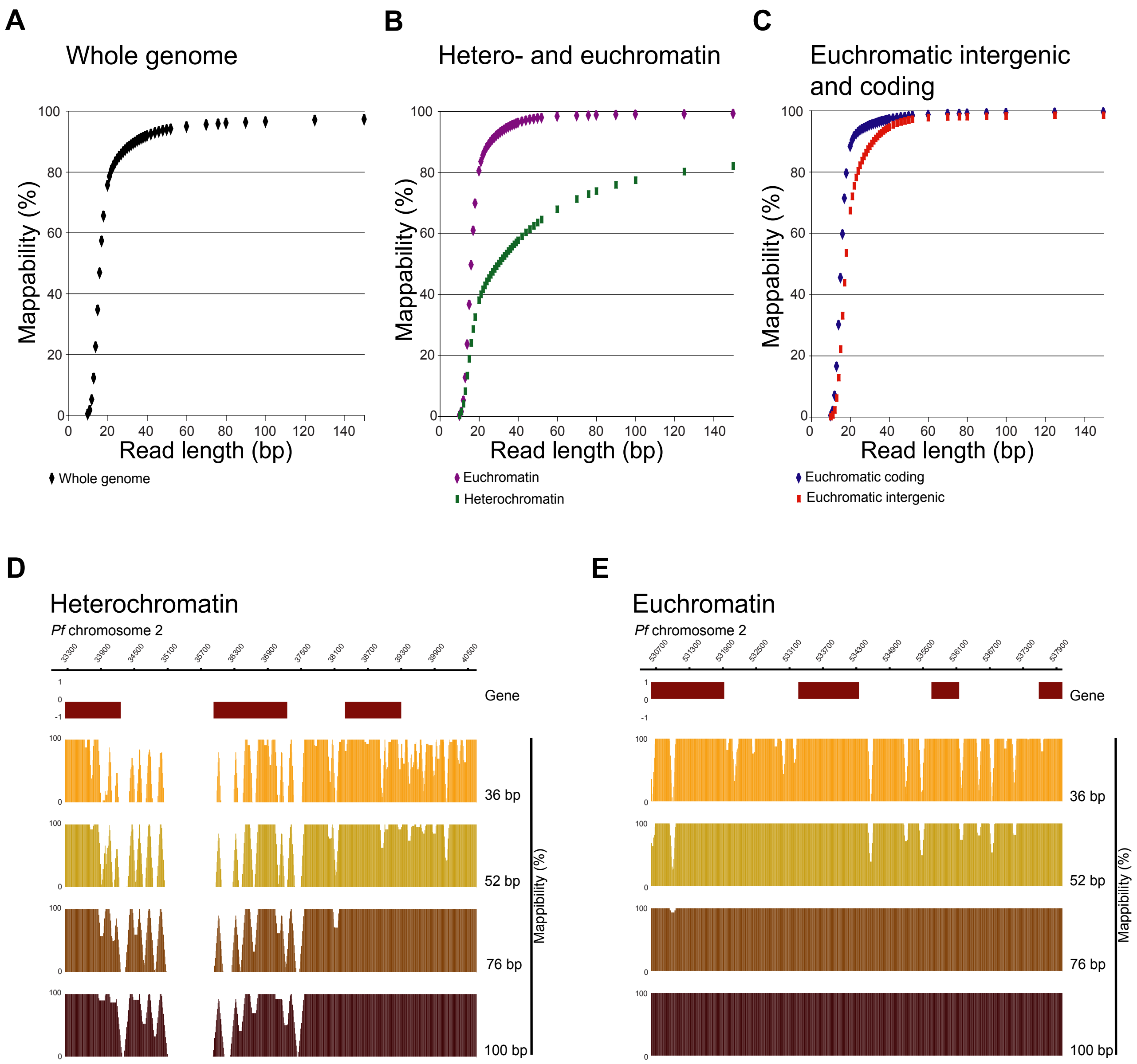
A
Whole genome
Mappability (%)
Read length (bp)
Whole genome
B
Hetero- and euchromatin
Mappability (%)
Read length (bp)
Euchromatin
Heterochromatin
C
Euchromatic intergenic and coding
Mappability (%)
Read length (bp)
Euchromatic coding
Euchromatic intergenic
D
Heterochromatin
Pf chromosome 2
Gene
36 bp
52 bp
76 bp
100 bp
Mappibility (%)
E
Euchromatin
Pf chromosome 2
Gene
36 bp
52 bp
76 bp
100 bp
Mappibility (%)
