# Supplementary figures and images for: H2A.Z Demarcates Intergenic Regions of the Plasmodium falciparum Epigenome That Are Dynamically Marked by H3K9ac and H3K4me3
Source: PLoS Pathog. 2010 Dec 16;6(12):e1001223. doi: 10.1371/journal.ppat.1001223 (PMC3002978; doi:10.1371/journal.ppat.1001223)

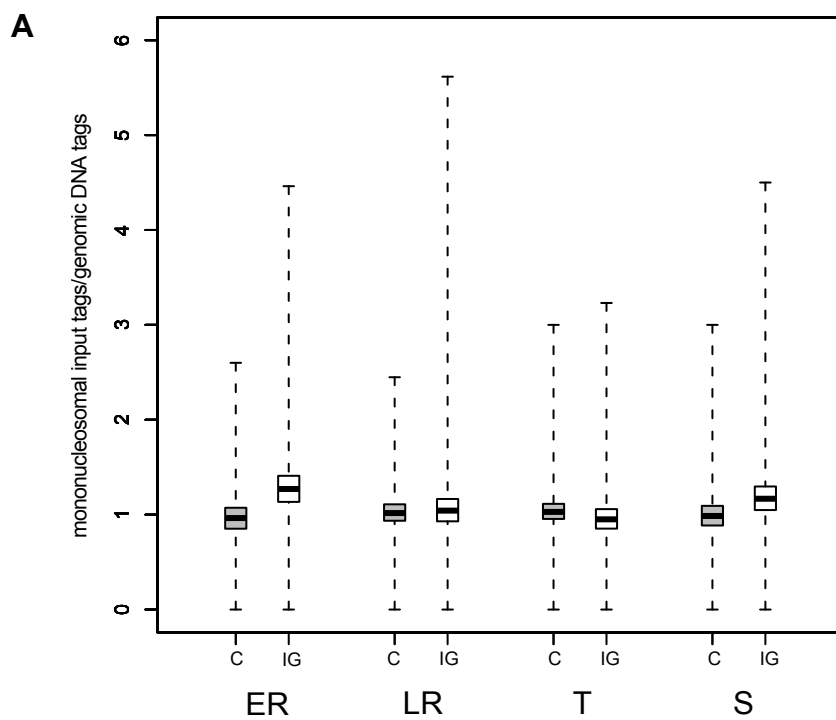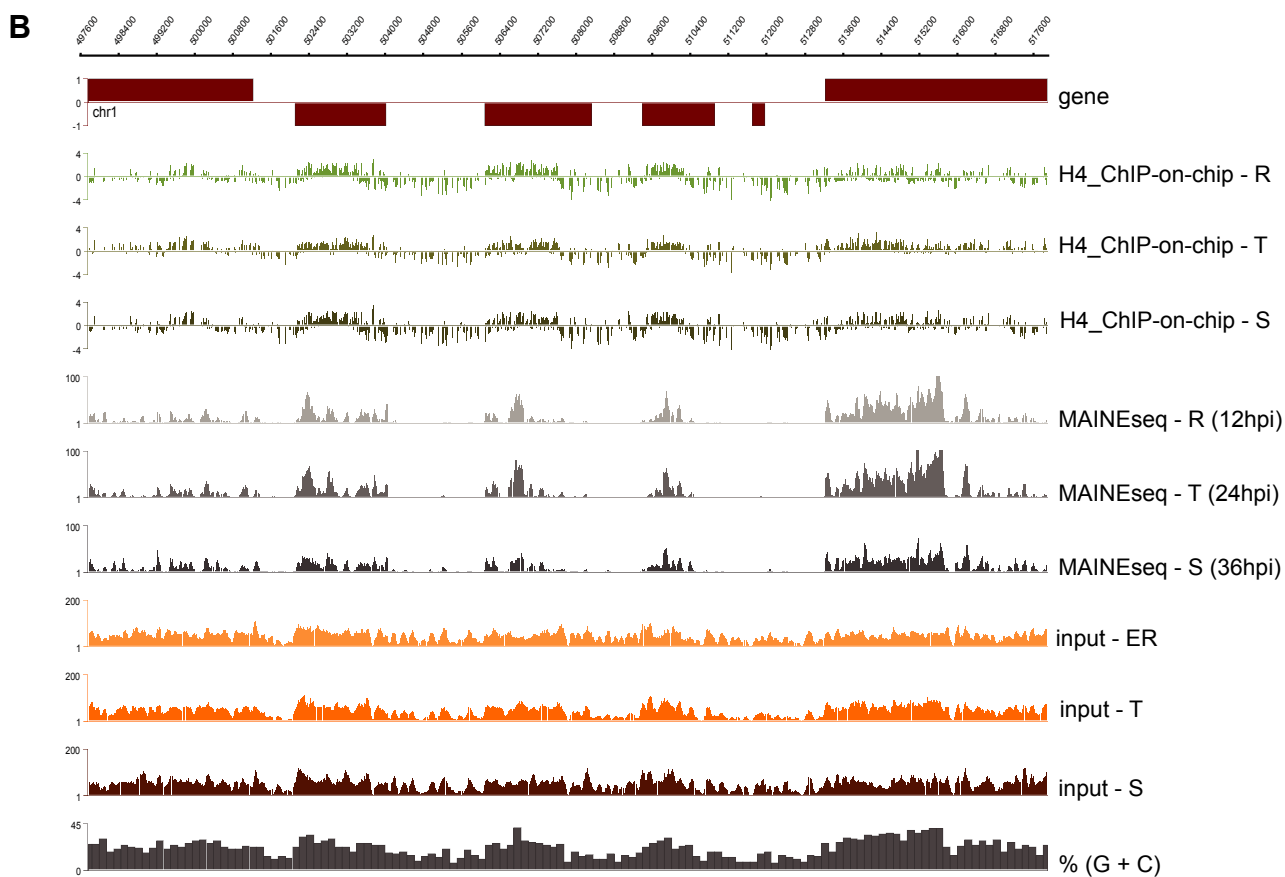

Westenberger et al., 2009

Ponts et al., 2010

this study

Supplement: Figure S3 — Nucleosome occupancy is similar between coding and intergenic regions. (A) Box-plot displaying the ratios of mono-nucleosomal vs genomic DNA tag counts in every coding (C) and intergenic (IG) region of the P. falciparum genome at four different stages of intra-erythrocytic development (ER: early ring; LR: late ring; T: trophozoite; S: schizont). (B) Representative screenshot showing direct comparison of H4_ChIP-on-chip (log2-ratio ChIP-over-genomic DNA) [31], MAINE-seq [30] and our mononucleosomal Illumina sequencing coverage plots at three stages of intra-erythrocytic development (ER/R: early ring/ring; T: trophozoite; S: schizont). Bottom track displays GC-content (%) per 150bp window. (0.72 MB PDF) [file ppat.1001223.s003.pdf]

**A**

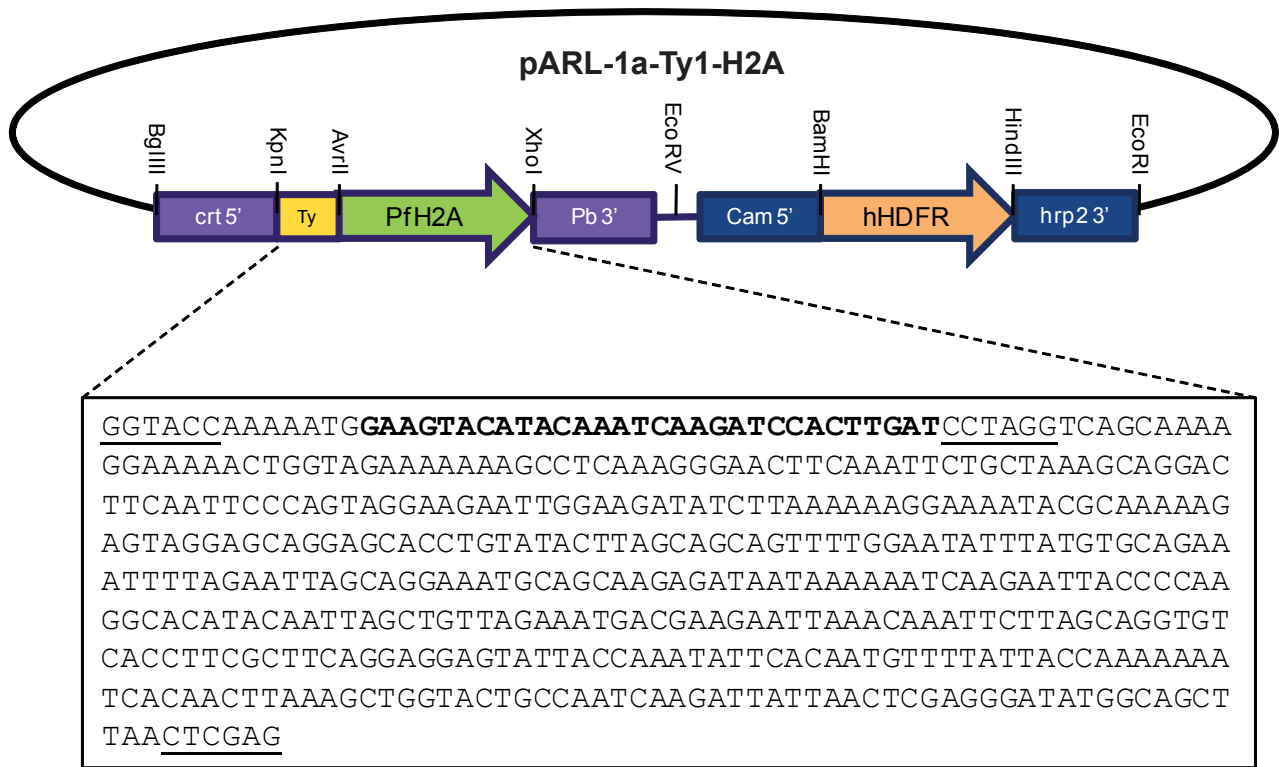

**B**

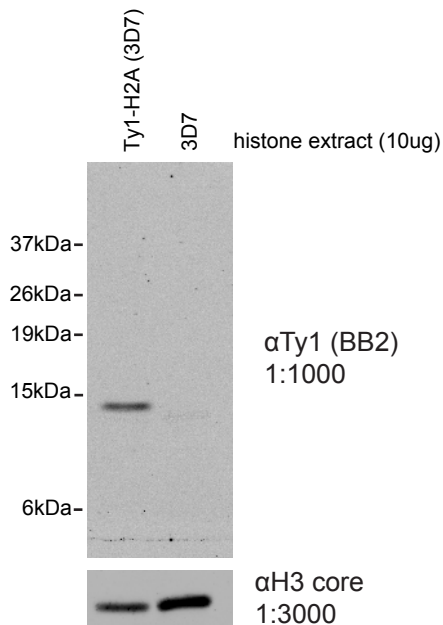

**C**

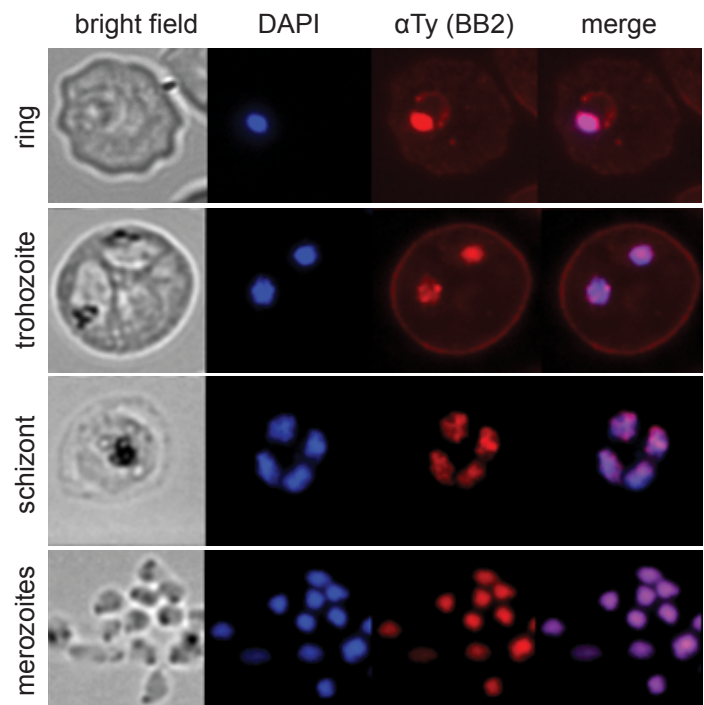

Supplement: Figure S6 — Generation and characterisation of H2A-Ty1 strain. (A) Map of the pARL-1a-Ty1-H2A plasmid transfected into 3D7 parasites. (B) Western blot demonstrating specific recognition of the Ty1-tagged H2A protein by the BB2 antibody in the transgenic parasite line. (C) Immunofluorescent assay showing nuclear localisation of the ectopically expressed Ty1-H2A protein at all stages of the intra-erythrocytic cycle. (0.95 MB PDF) [file ppat.1001223.s006.pdf]

## Average gene profile (Schizont)

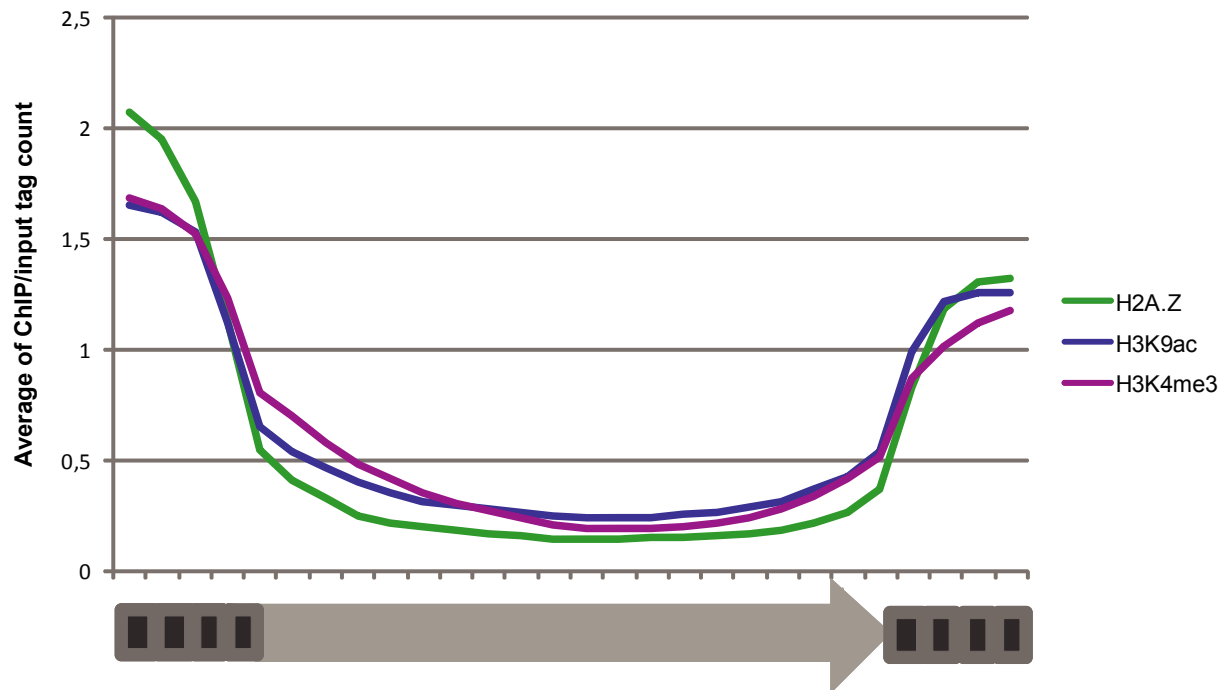

Supplement: Figure S7 — H3K9ac and H3K4me3 shows very similar localisation to H2A.Z. Average profile of H2A.Z occupancy, H3K9ac and H3K4me3 marking over euchromatic genes in schizont stage parasites. The coding region is indicated by the arrow (divided into 20 bins), 5′ and 3′ intergenic regions are displayed as 4 blocks of 150bp. (0.17 MB PDF) [file ppat.1001223.s007.pdf]

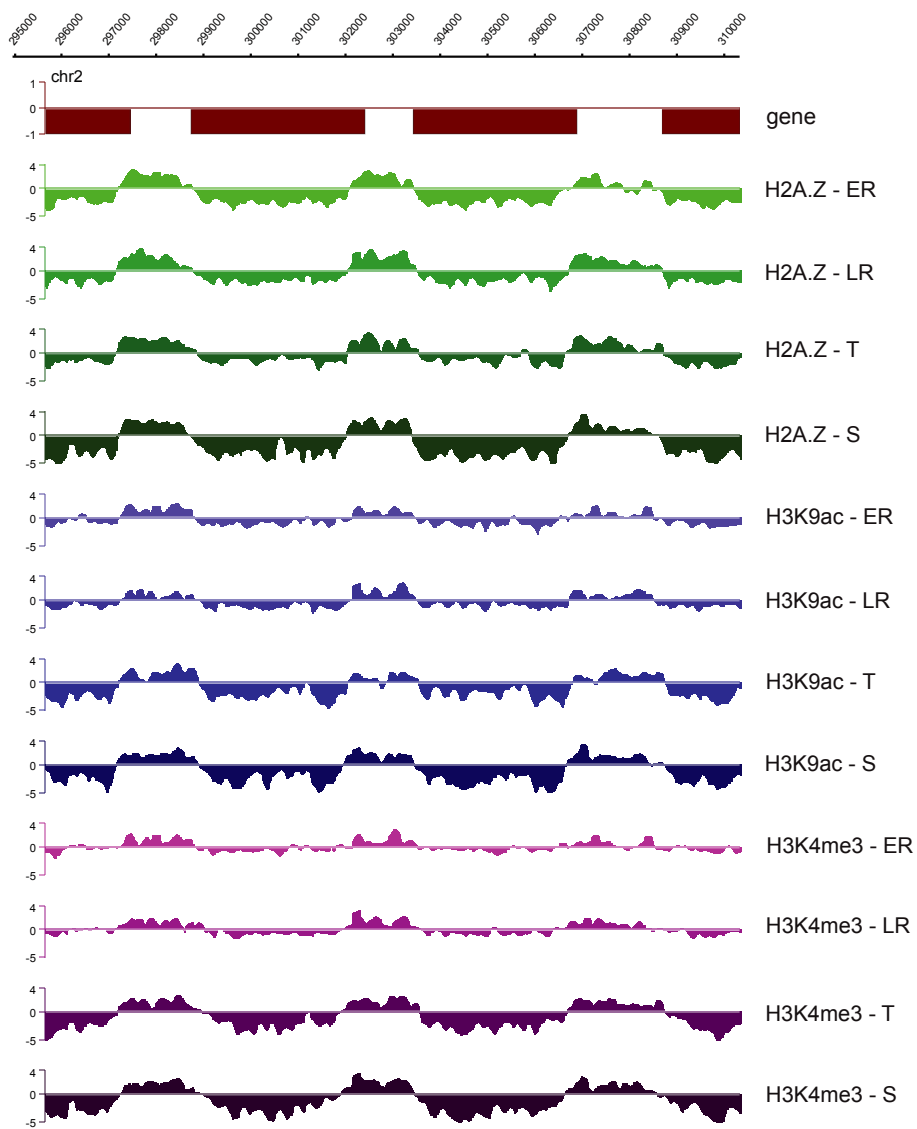

Supplement: Figure S8 — H2A.Z occupancy is invariable, while H3K9ac and H3K4me3 markings are dynamic through the intra-erythrocytic cycle. Screenshot of same chr2 region as shown in Figure 3. Instead of coverage plots, H2A.Z-, H3K9ac- and H3K4me3 over mono-nucleosomal input log2-ratios are displayed at four stages of intra-erythrocytic development (ER: early ring; LR: late ring; T: trophozoite; S: schizont). (0.49 MB PDF) [file ppat.1001223.s008.pdf]

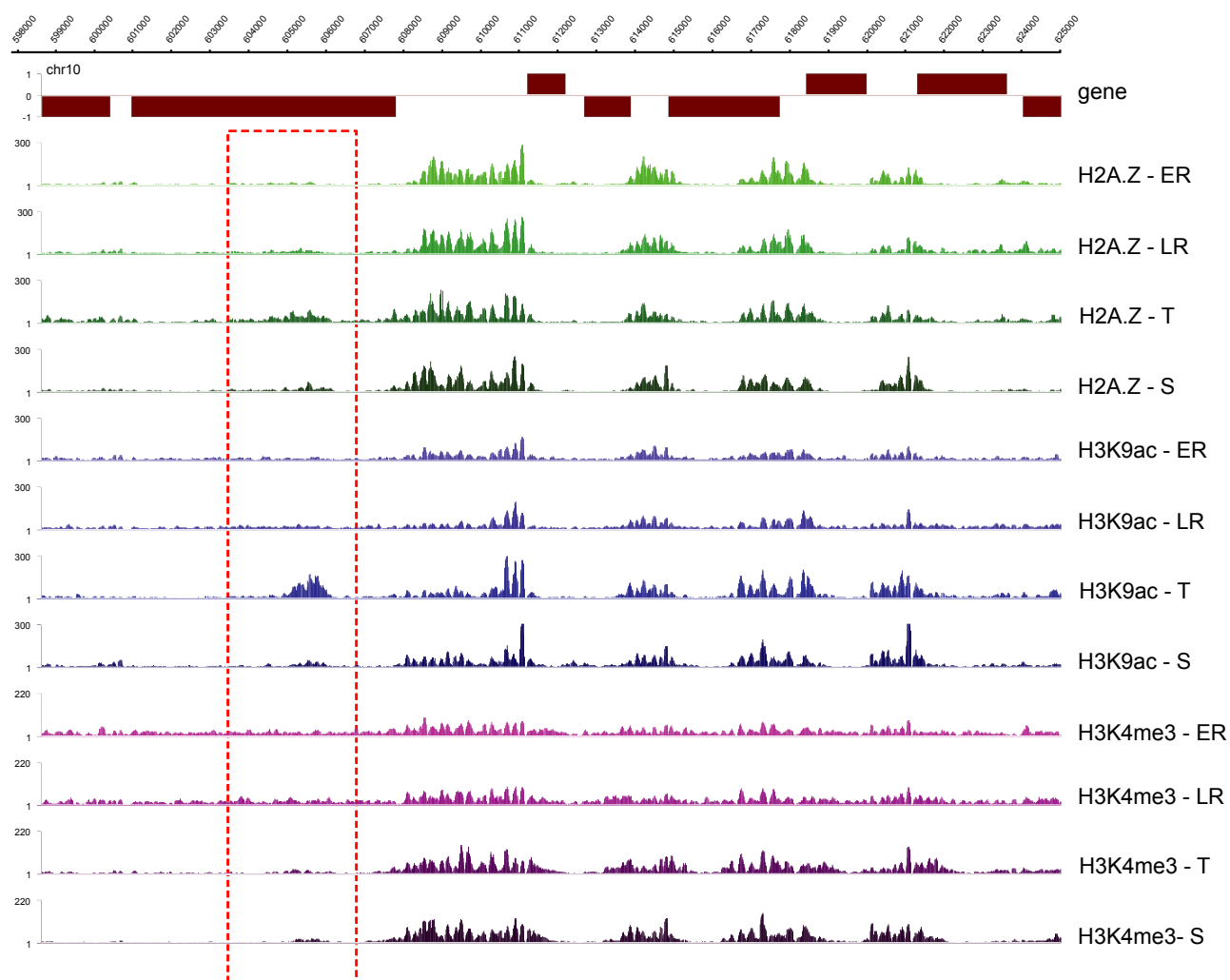

Supplement: Figure S9 — Regions specifically acetylated on H3K9 independent of H2A.Z during trophozoite stage, might corresponds to origins of replication. Screenshot of the H2A.Z, H3K9ac and H3K4me3 ChIP-seq coverage plots at four stages of intra-erythrocytic development (ER: early ring; LR: late ring; T: trophozoite; S: schizont) from a subsection of chromosome 10. The region with increased H3K9ac only in trophozoite stage is surrounded by a dashed line. (1.53 MB PDF) [file ppat.1001223.s009.pdf]

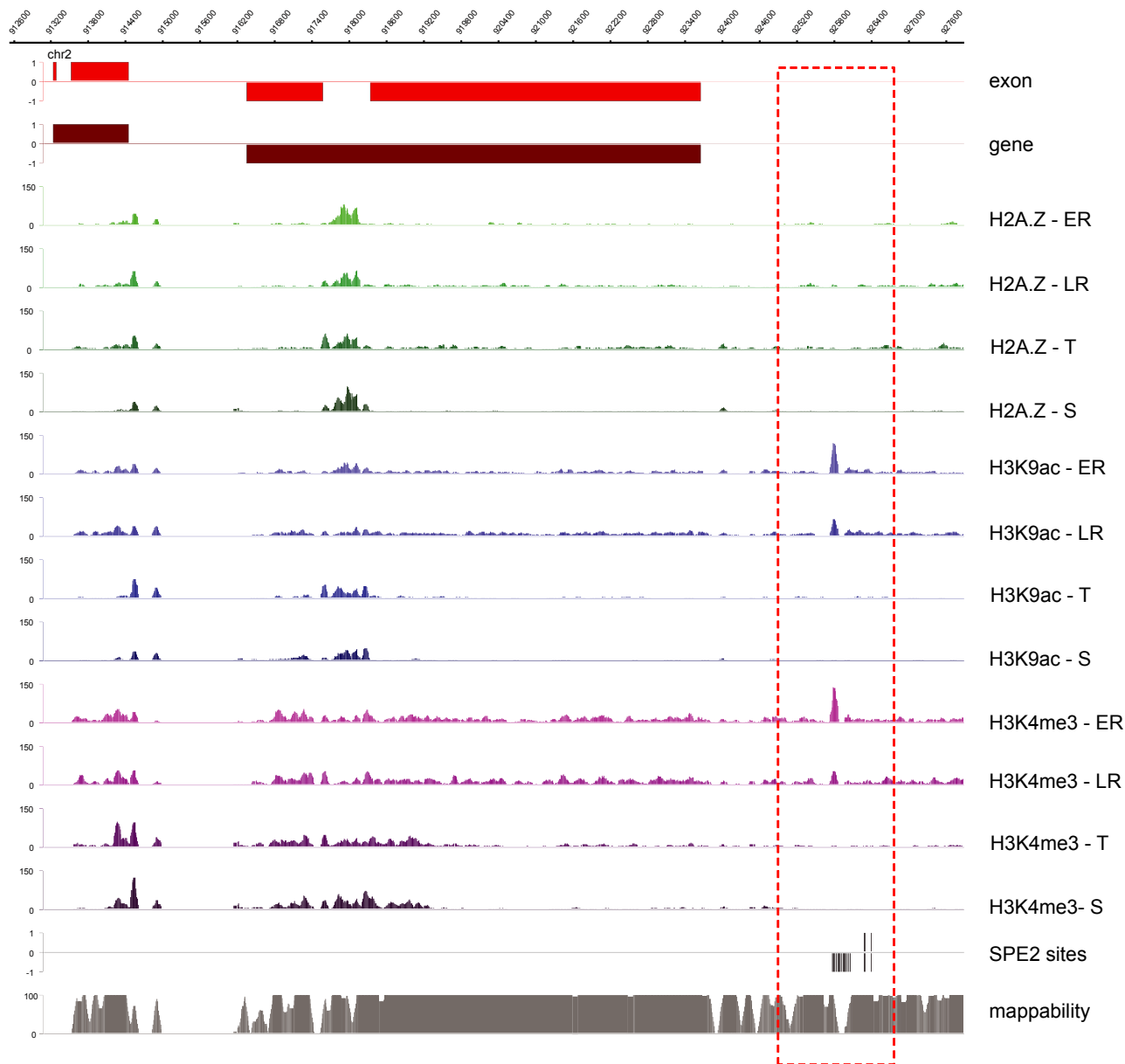

Supplement: Figure S10 — SPE2 repeats upstream of upsB type var genes carry H3K9ac and H3K4me3 independent of H2A.Z. Screenshot of the H2A.Z, H3K9ac and H3K4me3 ChIP-seq coverage plots at four stages of intra-erythrocytic development (ER: early ring; LR: late ring; T: trophozoite; S: schizont) from the distal end of chromosome 2. SPE2 repeat containing region carrying H3K9ac and H3K4me3 during ring stage is surrounded by a dashed line. (1.13 MB PDF) [file ppat.1001223.s010.pdf]

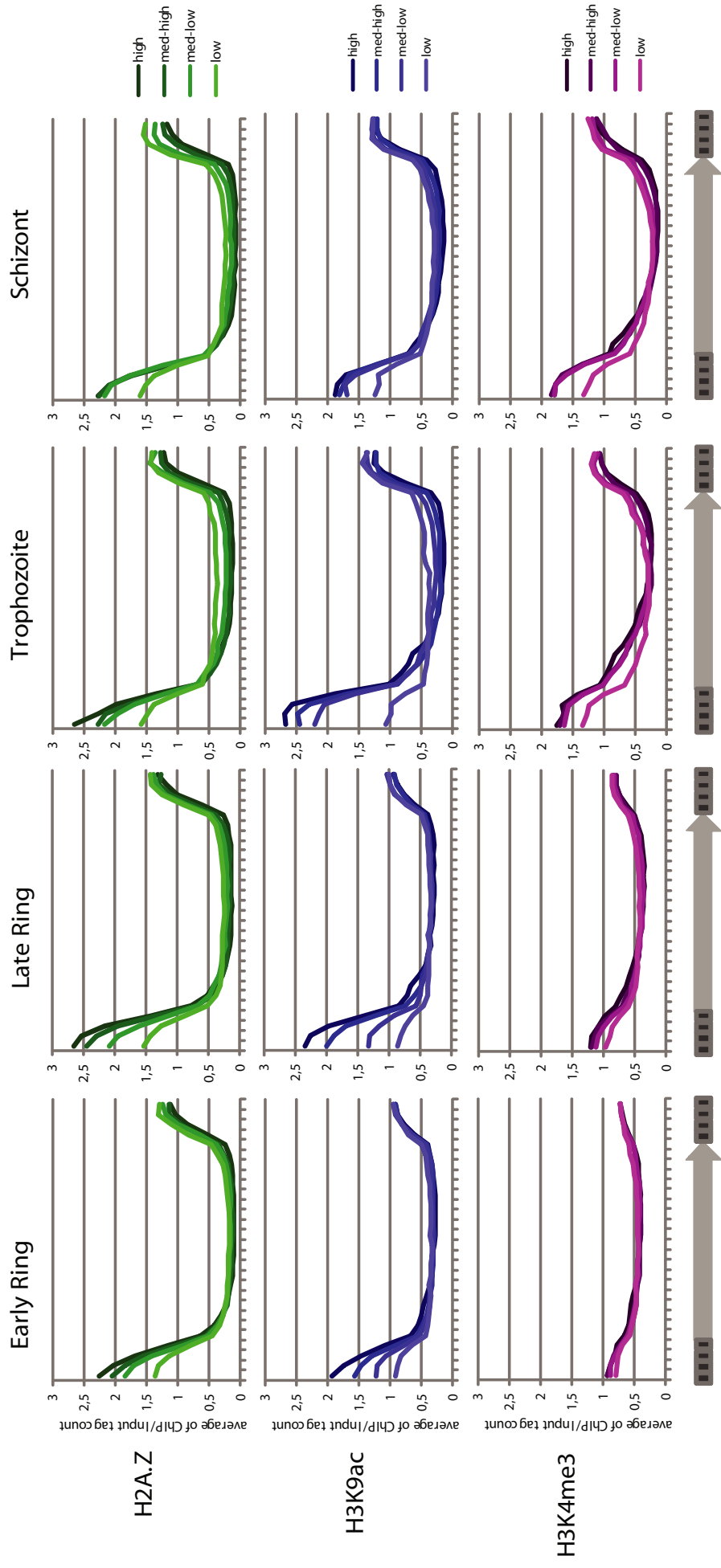

Supplement: Figure S11 — H2A.Z occupancy and H3K9ac marking upstream of genes correlates with steady state mRNA levels. Average profile of H2A.Z occupancy, H3K9ac and H3K4me3 marking over groups of genes with different mRNA levels at four stages of intra-erythrocytic development. To avoid signal coming from the neighbouring genes, only euchromatic genes (∼2600) with a minimum of 800bp intergenic region on both sides were included in this analysis. Coding body is indicated by the arrow (divided into 20 bins), 5′ and 3′ intergenic regions are displayed as 4 blocks of 150bp. (0.72 MB PDF) [file ppat.1001223.s011.pdf]
