# Supplementary material for: H2A.Z Demarcates Intergenic Regions of the Plasmodium falciparum Epigenome That Are Dynamically Marked by H3K9ac and H3K4me3
Source: PLoS Pathog. 2010 Dec 16;6(12):e1001223. doi: 10.1371/journal.ppat.1001223 (PMC3002978; doi:10.1371/journal.ppat.1001223)
Supplement: Figure S4 — RNA-seq interdataset comparison and scaling method. (A) R2-correlation values from pair-wise comparison of our 8 stages RNA-seq and 7 stages of published RNA-seq data [4] of 3D7 Plasmodium falciparum parasites. Colour code indicates the level of correlation (black = highest, white = lowest). (B) Graph showing the scaling factor used for scaling of RNA-seq data to better approximate transcriptional activity per nucleus. Scaling factor is calculated based on the amount of total RNA collected from each stage, divided by the average number of nuclei per parasite in that stage. (0.23 MB PDF) [file ppat.1001223.s004.pdf]

A

		Otto *et al.*, 2010							This study							
		0 hpi	*8 hpi*	*16 hpi*	*24 hpi*	*32 hpi*	*40 hpi*	*48 hpi*	*5 hpi*	*10 hpi*	*15 hpi*	*20 hpi*	*25 hpi*	*30 hpi*	*35 hpi*	*40 hpi*
Otto *et al.*, 2010	*0 hpi*	1.0000														
	8 hpi	0.9225	1.0000													
	16 hpi	0.8077	0.8870	1.0000												
	24 hpi	0.7419	0.7543	0.8167	1.0000											
	32 hpi	0.7030	0.6289	0.6335	0.8303	1.0000										
	40 hpi	0.7353	0.6363	0.5252	0.6422	0.7867	1.0000									
	48 hpi	0.7725	0.6939	0.5715	0.6319	0.7219	0.8839	1.0000								
This study	*5 hpi*	0.7035	0.6856	0.5339	0.4163	0.3718	0.5236	0.5729	1.0000							
	10 hpi	0.7086	0.7792	0.6975	0.5237	0.3882	0.4167	0.4755	0.8426	1.0000						
	15 hpi	0.6331	0.7503	0.7617	0.5825	0.3883	0.3255	0.3690	0.6521	0.8927	1.0000					
	20 hpi	0.5795	0.6911	0.7750	0.6603	0.4348	0.3093	0.3280	0.5359	0.7768	0.9126	1.0000				
	25 hpi	0.5410	0.6018	0.7039	0.7404	0.5595	0.3524	0.3263	0.4414	0.6184	0.7375	0.8659	1.0000			
	30 hpi	0.5564	0.5598	0.6202	0.7283	0.6760	0.4439	0.3716	0.4311	0.5433	0.6093	0.7165	0.9032	1.0000		
	35 hpi	0.5925	0.5478	0.5600	0.6658	0.7345	0.5616	0.4369	0.4744	0.5208	0.5319	0.5999	0.7617	0.9079	1.0000	
	40 hpi	0.6331	0.5515	0.4931	0.5666	0.7107	0.7147	0.5702	0.5888	0.5293	0.4549	0.4635	0.5594	0.7076	0.8722	1.0000

0.2-0.4
0.4-0.6
0.6-0.8
0.8-1
1.0000

B

Correction of RNA-seq based on amount of total RNA per nucleus

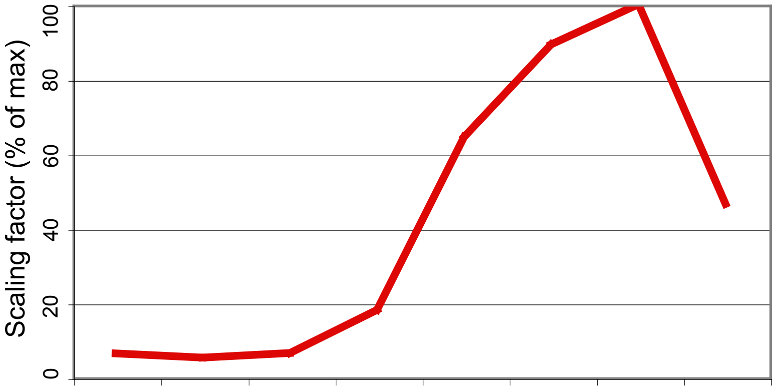

	5hpi	10hpi	15hpi	20hpi	25hpi	30hpi	35hpi	40hpi
Total RNA (ug)	9	4	5	14	50	96	209	195
nuclei/parasite	1.83	1	1	1	1	1.39	2.69	5.42
Scaling factor	0.0633	0.0515	0.0644	0.1802	0.6435	0.8921	1	0.4635
