# Supplementary material for: H2A.Z Demarcates Intergenic Regions of the Plasmodium falciparum Epigenome That Are Dynamically Marked by H3K9ac and H3K4me3
Source: PLoS Pathog. 2010 Dec 16;6(12):e1001223. doi: 10.1371/journal.ppat.1001223 (PMC3002978; doi:10.1371/journal.ppat.1001223)

A

```
PfH2A.Z    -------------------------MEVPGKVIGGKVGGKVGGKVLGLGKGGKGKTGSGKT-K   38
TgH2A.Z    -------------------------MDGAGKV-GGKVGGKVGGKVGGMGKGGKGKSGSGKG-K   37
HsH2A.Z    ------------------------------------------------MAGGKAGKDSGKA-K   15
TbH2A.Z    MSLTGDDAVPQAPLVGGVAMSPEQASALTGGKLGGKAVGPAHGKGKGKGKGKRGGKTGGKAGR   63
PfH2A      ------------------------------------------------MSAKGKTGRKKASK   14
TgH2A      ------------------------------------------------MSAKGAGGRKKTSS   14
HsH2A      ------------------------------------------------MSGRGKQGGKARAK   14
TbH2A      -------------------------------------------------MATPKQAVKKASK   13

PfH2A.Z    KAPLSRASRAGLQFPVGRVHRMLKSRISSDGRVGSTAAVYAAAILEYLTAEVLELAGNATKDL  100
TgH2A.Z    KAPLSRAARAGLQFPVGRVHRMLKSRISSEGRVGSTAAVYASAILEYLTAEVLELAGNASKDL   99
HsH2A.Z    TKAVSRSQRAGLQFPVGRIHRHLKSRTTSHGRVGATAAVYSAAILEYLTAEVLELAGNASKDL   77
TbH2A.Z    RDKMTRAARADLNFPVGRIHSRLKDGLNRKQRCGASAAIYCAALLEYLTSEVIELAGAAAKAQ  126
PfH2A      G--TSNSAKAGLQFPVGRIGRYLKKGKYAK-RVGAGAPVYLAAVLEYLCAEILELAGNAARDN   74
TgH2A      GKKVSRSAKAGLQFPVSRIGRYLKKGRYAK-RVGVGAPVYLAAVLEYLCAEILELAGNAARDH   76
HsH2A      A--KTRSSRAGLQFPVGRVHRLLRKGNYAE-RVGAGAPVYLAAVLEYLTAEILELAGNAARDN   74
TbH2A      GG-SSRSVKAGLIFPVGRVGTLLRRGQYAR-RIGASGAVYMAAVLEYLTAELLELSVKAAAQQ   74
                :.: :*.* ***.*:    *:        * *   ..:* :*:**** :*::**:*:

PfH2A.Z    --KVKRITPRHLQLAIRGDEELDTLIK-ATIAGGGVIPHIHKALMNKVPLPPTAQKKPKKN    158
TgH2A.Z    --KVKRITPRHLQLAIRGDEELDTLIK-ATIAGGGVIPHIHKSLMTKGPSTQPMKKAKK--    155
HsH2A.Z    --KVKRITPRHLQLAIRGDEELDSLIK-ATIAGGGVIPHIHKSLIGKKGQQKTV-------    128
TbH2A.Z    --KTERIKPRHLLLAIRGDEELNQIVN-ATIARGGVVPFVHKSLEKKIIKKSKRGS-----    179
PfH2A      --KKSRITPRHIQLAVRNDEELNKFLAGVTFASGGVLPNIHNVLLPKKSQLKAGT-ANQDY    132
TgH2A      --KKTRIIPRHIQLAVRNDEELSKFLGGVTIANGGVMPHVHAVLLPKHSKSKGKHGVSQEF    135
HsH2A      --KKTRIIPRHLQLAIRNDEELNKLLGKVTIAQGGVLPNIQAVLLPKKTESHHKAKGK---    130
TbH2A      TKKTKRLTPRTVTLAVRHDDDLGALLRNVTMSRGGVMPSLNKALAKKQKSGKHAKATPSV-    134
             *   *: **  :  **:*  *::*. ::  .*::  ***:*  ::   *   *
```

B

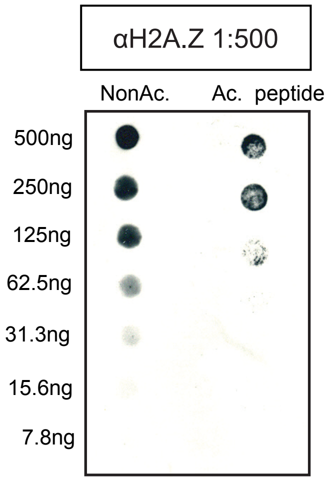

C

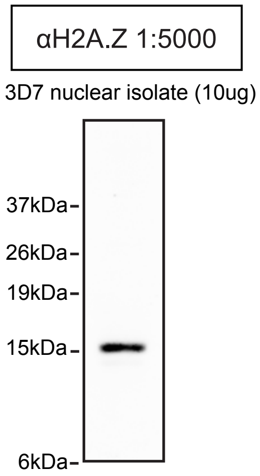

Supplement: Figure S5 — Generation and characterisation of polyclonal H2A.Z antibody. (A) Alignment of the protein sequence of histone H2A and H2A.Z proteins from H. sapiens, P. falciparum, T. brucei, T. gondii. The highly conserved core domain is surrounded by a dashed line. Acetylated lysines at the N-terminus of H2A.Z are bolded and underlined. The peptide used for immunisation is highlighted in yellow. (B) Dot blot experiment demonstrating similar affinity of the H2A.Z antibody to the acetylated and non-acetylated peptide. (C) The H2A.Z antibody specifically recognises its target protein in total nuclear isolate from asynchronous parasite culture. (0.91 MB PDF) [file ppat.1001223.s005.pdf]
